# Spatial Cell Atlas of Lateral Septum Reveals Changes Underlying Anxiety and Fear Learning Deficits in Mice with Abnormal Immunity

**DOI:** 10.7150/ijbs.117249

**Published:** 2025-10-01

**Authors:** Yian Wang, Wenxia Gao, Xueyan Yang, Zirui Liu, Hisham Al-Ward, Orion R Fan, Yiming Shao, Liqiang Zhou, Bo Jing, Qianxiang Wu, Wenmin Zhu, Wei Chen, Yi Eve Sun

**Affiliations:** 1 Stem Cell Translational Research Center, Tongji Hospital, School of Medicine, Tongji University, Shanghai, 200065, China; 2 Faculty of Life and Health Sciences, Shenzhen University of Advanced Technology (SUAT), Shenzhen, Guangdong 518000, China; 3 Brain Cognition and Brain Disease Institute, Shenzhen Institutes of Advanced Technology, Chinese Academy of Sciences, Shenzhen 518055, China

**Keywords:** Lateral septum (LS), anxiety and fear, immune-imbalance, single-nucleus RNA sequencing (snRNA-seq), estrogen receptor 1 (*Esr1*), three-dimensional atlas.

## Abstract

The lateral septum (LS) regulates affective-cognitive processes and is dysregulated in human psychiatric disorders. Its functional complexity stems from heterogeneous connectivities and neuronal subtype compositions across anatomical subregions. Comprehensive understanding of LS function has been hindered by the lack of a three-dimensional (3D) neuronal atlas. Moreover, the association between peripheral immune disturbances and psychiatric disorders underscores the necessity to investigate potential 3D LS neuronal heterogeneities across distinct immunological states. This study employed single-nucleus RNA sequencing to analyze LS differences in C57J, BALB/c, and nude mice with distinct immune contexts (i.e., immunocompetent, enhanced Th2 reactivity, and T-cell deficiency, respectively), and identified global alterations in non-neuronal cells alongside with neuron-specific changes. Through combining publicly available multiplexed error-robust fluorescence *in situ* hybridization (MERFISH) data with neural circuit tracing, this study further constructed a 3D neuronal atlas of the entire LS with their projection connectivities. Notably, an *Esr1*-positive neuronal subpopulation distributed in the ventral LS exhibited potential responsiveness to changes in peripheral immunity and may participate in anxiety regulation. Furthermore, the dorsal LS demonstrated heterogeneity in fear memory regulation associated with T cell homeostasis. These findings underscore the critical role of immune-neural crosstalk in emotional regulation while shedding light on potential therapeutic targets for emotion-related disorders linked to T-cell homeostatic disturbances.

## Introduction

The septum serves as a critical hub in the limbic system, regulating emotional responses, social functions, and reproductive behaviors^1^,^2^. In mice, the septum is primarily divided into the medial septum (MS) and lateral septum (LS). In primates, the septum exhibits a higher degree of cortical connectivity specialization^3^,^4^, reflecting its evolutionary expansion from supporting basic survival mechanisms to enabling complex social adaptation. Among these subregions, the LS functions as an integration hub for emotion-motivation processing and is closely linked to human neuropsychiatric disorders such as anxiety, bipolar disorder, autism spectrum disorders, schizophrenia, and post-traumatic stress disorder (PTSD)^5^. Investigating the evolutionary conservation of emotional circuits in mice provides a critical foundation for deciphering complex human emotional networks and developing therapeutic interventions.

The regulation of emotional responses mediated by the LS in mice exhibits significant heterogeneity, closely linked to its complex structural organization^6^. Initially, based on anatomical morphology, and Nissl staining, the LS was simply divided into dorsal (LSd), intermediate (LSi), and ventral (LSv) subregions^7^. This classification provided the earliest spatial framework and served as the foundation for all subsequent studies. However, this approach showed weak correlation with functional regulation. Immunohistochemical detection of various neuropeptides later revealed cellular heterogeneity and more detailed identification of the rostral LS (LSr) along with medial-lateral subdivisions^8^. Nevertheless, accumulating evidence demonstrated that neurons expressing the same neuropeptide (e.g., somatostatin (SST)) exhibit distinct spatial and functional heterogeneity^9^, thereby hindering precise functional and projection analyses of specific neuronal subpopulations. Recent studies have begun applying single-nucleus RNA sequencing (snRNA-seq) to more precisely define neuronal subpopulations^5^,^10^-^12^, yet spatial information has remained limited, particularly within a three-dimensional spatial framework.

The suppression of anxiety and fear was initially recognized as one of the key functions of the LS. Lesions or reversible inactivation of the septum (including MS and LS) led to a phenotype called “septal rage”, characterized by an exaggerated defensive response to non-threatening stimuli^13^. Activation of SST-positive neurons in the LSd exhibited anti-anxiety functions^14^,^15^. Whereas, in LSi, projections from corticotropin-releasing hormone receptor 2 (CRHR2) neurons to anterior hypothalamic nucleus (AHN) mediated anxiety potentiation^13^. Furthermore, the LSv exhibited even greater heterogeneity in anxiety regulation upon receiving projection input^6^. Although fear and anxiety share overlapping circuit mechanisms^16^, studies that associated fear with the LS have primarily focused on the ​LSd. Projections from ​dorsal CA1 (CA1.d) and the ​subiculum nucleus to the ​medial LSd enhanced fear, while projections from dorsal CA3 (CA3.d) inhibited fear^6^. These observations indicated that the regulation of anxiety/fear by the LS involves ​region-specific, neuron subtype-specific, and projection-specific mechanisms.

Compared to state anxiety, trait anxiety manifests as persistent and pathological, typically reflecting stable alterations in neural circuit composition or signaling intensity^17^,^18^. However, it is surprisingly notable that the state of the peripheral circulating system is also intricately linked to trait anxiety regulation. Originally, research found that immunodeficient mice had developmental deficits in neural synapses and exhibited anxiety or depressive behavior^19^, which was also confirmed in T cell knockout mice^20^,^21^. BALB/c mice have also been described as having increased anxiety, which has sparked interest in mechanistic investigation of T helper 2 cell (Th2)-skewed anxiety, as the Th2 response and CD4-T cell count in BALB/c mice is abnormally higher than that in wildtype C57J mice^22^-^24^. Patients with Th2 driven atopic dermatitis and asthma typically exhibit a higher incidence of anxiety, and their Th2 proportion is higher compared to patients with the same underlying disease but without trait anxiety^25^,^26^. Additionally, anxiety patients with 22q11.2 deficiency syndrome (thymic hypoplasia) exhibit an increased number of CD4-T cells^27^,^28^. Similarly, peripheral immune disturbances have been shown to be highly correlated with fear memory^29^. Although these data can demonstrate the relationship between peripheral immunity and emotion regulation, the understanding of how immune deficiency or Th2 enhancement changes neural circuits is still insufficient.

In this study, snRNA-seq was performed on the LS of wildtype C57J, BALB/c, and nude mice with distinct immune backgrounds, comparative analyses were conducted between non-neuronal and neuronal populations. Based on neuronal subpopulation transcriptomic signatures, tissue sections spanning the medial-lateral (X)-, rostral-caudal (Y)-, and dorsal-ventral (Z)-axes, encompassing the entire LS, were selected to spatially map these subpopulations in three spatial dimensions and differences in their respective circuit projection networks were investigated. Additionally, the evolutionary conservation between humans and mice was explored. Our findings revealed a specific neuronal subpopulation within the LSv that likely regulates anxiety-like behaviors and exhibits potential sensitivity to perturbations in immune and hormone states. Furthermore, a potential regulatory role of dorsal neuron populations in fear memory was identified. This work not only established a spatial neuronal atlas of the entire LS, but also provided novel insights that will inform future therapeutic strategies for emotional disorders via peripheral interventions.

## Results

### Increased anxiety and associated myelin deficiency in nude and BALB/c mice

**Figure 1:**
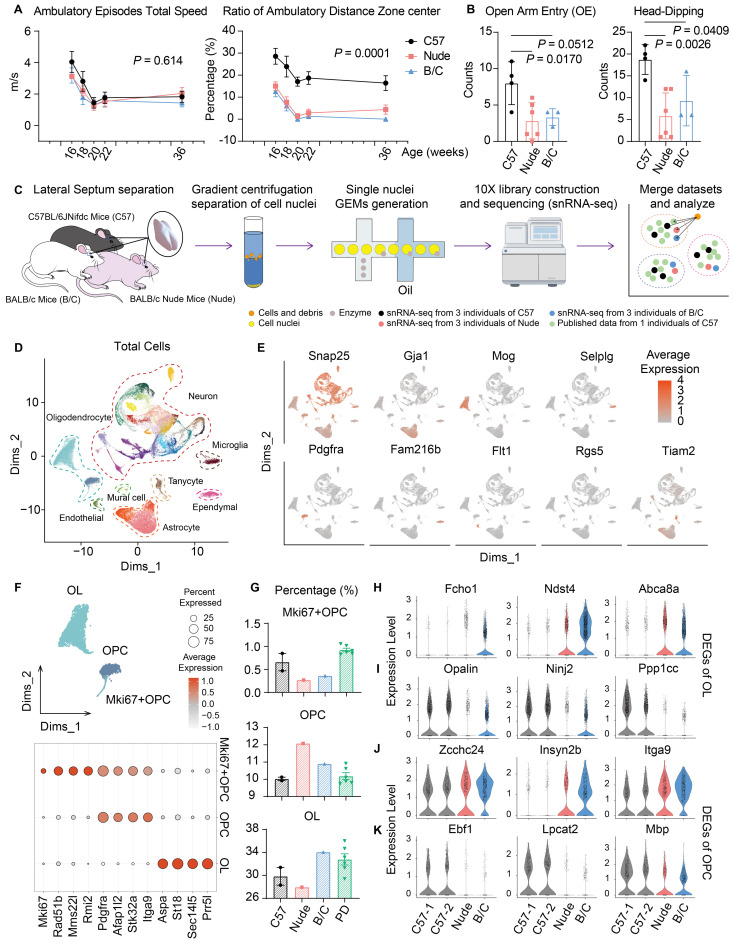
** Increased anxiety and associated myelin deficiency in nude and BALB/c mice. (A)** Open field test, (C57J, n = 6; nude, n = 6; BALB/c, n = 6; repeated measure ANOVA with Bonferroni posttest was used). **(B)** Elevated plus maze test, (C57J, n = 4; nude, n = 6; BALB/c, n = 3; Student's t test was used). **(C)** Schematic of the snRNA-seq of the LS, (C57J, merge 6 individual mice into 2 samples; nude, merge 3 individual mice into 1 sample; BALB/c, merge 3 individual mice into 1 sample). **(D)** Integrated UMAP of here and public snRNA-seq data, showing 8 general cell type annotations according the canonical marker. **(E)** Feature plots show maker gene expression of 8 major clusters. **(F)** The UMAP plot illustrates 3 subtypes of oligodendrocyte lineage. Dot plots were employed to show marker genes of these 3 subtypes (OL: oligodendrocyte; OPC: oligodendrocyte precursor cell). **(G)** Proportions of 3 oligodendrocyte subtypes across three mouse strains (PD: public dataset of C57J mice). **(H-K)** Representative DEGs in OL/OPC subtypes across C57J, nude, and BALB/c mice. All data are expressed as mean ± SEM.

In open-field assays, both nude mice (T-cell deficient from BALB/c background) and BALB/c mice (Th2 enhancement) exhibited reduced ambulatory distance in the central area of the open field without impaired motor ability (Fig. 1A). The same anxiety-related phenotype was also observed in the elevated plus maze test (Fig. 1B). Similar to previous research findings^22^, these results suggest a potential role of T-cell homeostasis in regulating anxiety. To investigate potential perturbations in the lateral septum (LS) under distinct immune backgrounds, intact LS tissues were collected from C57J, BALB/c, and nude mice, followed by nuclei extraction via high-speed gradient centrifugation and subsequent sequencing. During data analysis, published single-nucleus RNA sequencing (snRNA-seq) dataset of LS from C57J mice was incorporated for sensitivity validation of cellular subpopulation identification (Fig. 1C)^10^. Uniform Manifold Approximation and Projection (UMAP) revealed 41 transcriptionally distinct clusters, which were categorized into the following 8 major cell classes based on canonical marker gene expressions (Fig. 1D, E; Fig. S1A). To enhance the effectiveness of sensitivity analysis, the preference differences were identified between our sequencing data and public data. The results demonstrated that the nuclei isolation method employed here exhibited enhanced neuronal preservation, whereas non-neuronal populations—particularly, mural cells, endothelial cells, and ependymal cells were potentially underrepresented (Fig. S1B-D). These three cell types were thus excluded from subsequent pipelines (Fig. S1E).

Changes in glial cells were first observed. In the oligodendrocyte lineage, cells were classified into mature oligodendrocytes (OLs), oligodendrocyte precursor cells (OPCs), and proliferating OPCs (Fig. 1F). Both nude mice and BALB/c mice exhibited reduced proliferative subpopulations (Fig. 1G). Notably, nude mice displayed characteristic impairments in oligodendrocyte maturation (Fig. 1G). Subsequent differentially expressed gene (DEG) analyses of OLs and OPCs revealed many consistent transcriptional alterations of nude and BALB/c mice, compared to C57J mice (Fig. S1F, G). In OLs, genes related to synaptic regulation (*Fcho1*), extracellular matrix (ECM) regulation (*Ndst4*), and myelin lipid metabolism (*Abca8a*) were upregulated, while genes related to myelination (*Opalin*), neuroimmune repair (*Ninj2*), and cell cycle regulation (*Ppp1cc*) were downregulated (Fig. 1H, I). In OPCs, genes related to ECM and cell migration (*Zcchc24*, *Insyn2b*, *Itga9*) were upregulated, while genes related to myelin formation and regeneration (*Ebf1*, *Lpcat2*, *Mbp*) were downregulated (Fig. 1J, K). Together these data are indicative of abnormal myelination in nude and BALB/c mice. Although there may be inherent differences in the genetic backgrounds of C57J and BALB/c, the enhancement of Th2 or T cell deficiency during developmental stages has been shown to lead to myelination and oligodendrocyte differentiation disorders^30^-^33^.

### Altered function and morphology of astrocytes in nude and BALB/c mice

**Figure 2:**
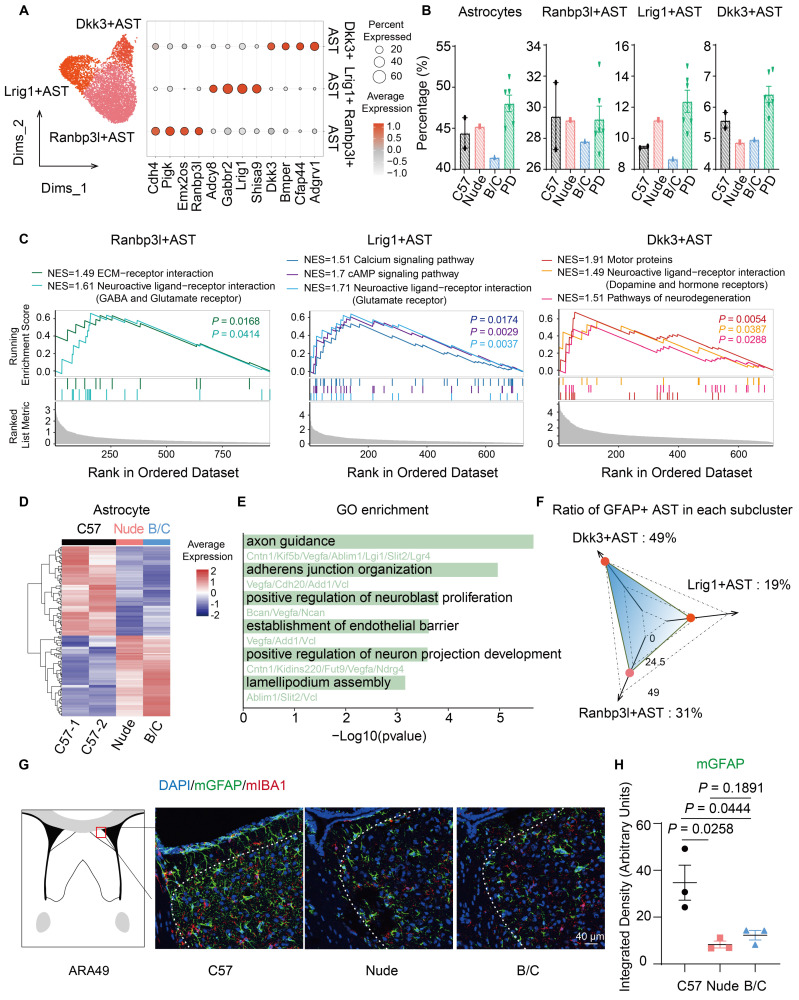
** Altered function and morphology of astrocytes in nude and BALB/c mice. (A)** The UMAP plot presents 3 subclusters of astrocytes, marking with *Dkk3*, *Lrig1* and *Ranbp3l*, respectively. Other marker genes were illustrated by the dot plot. **(B)** Proportions of total astrocytes and subclusters across mouse strains. **(C)** GSEA showed significantly enriched signaling pathways of subclusters. **(D)** Heatmap of DEGs in total astrocytes between C57J mice cohort and nude, BALB/c mice cohort. **(E)** Enriched GO terms of downregulated genes in nude and BALB/c mice, compared to C57J mice. **(F)** Radar plot of GFAP-positive cell percentage in each astrocyte subcluster. **(G)** Immunofluorescent staining of GFAP and IBA1 in mouse LS. **(H)** Quantitative analysis of the fluorescence intensity of GFAP-positive signals (mean ± SEM, n = 3). Student's t test was used.

Astrocyte subclusters were identified and marked by *Dkk3*, *Lrig1*, and *Ranbp3l* (Fig. 2A). Although only BALB/c mice demonstrated a relatively low proportion of all astrocytes, the proportions of *Dkk3*-positive astrocytes in nude mice and BALB/c mice significantly decreased (Fig. 2B). GSVA (Gene Set Variation Analysis) and GSEA (Gene Set Enrichment Analysis)-based functional annotation revealed that these three astrocyte subclusters exhibit distinct abundance levels of neurotransmitter receptor expression. Specifically, the *Dkk3*-positive astrocytes predominantly express dopamine receptors and hormone receptors (Fig. 2C; Fig. S1H), suggesting their potential involvement in emotional modulation, and environmental responsiveness^34^,^35^. Additional DEG analysis of astrocytes suggests a decrease in tight junctions and lamellipodium assembly in nude mice and BALB/c mice (Fig. 2D, E). This prompted further investigations into astrocytic morphological changes. Considering that GFAP has heightened expression in *Dkk3*-positive astrocytes (49%), immunofluorescent staining of GFAP was performed in the LS across the three mouse strains (Fig. 2F, G). Notably, astrocytes in C57J mice exhibited more pronounced branching morphology compared to other groups (Fig. 2G, H).

The limited characterization of microglia by snRNA-seq compromised the accuracy of proportional analysis. However, statistical analysis of immunofluorescence staining suggested that both nude mice and BALB/c mice exhibited reduced regional distribution of microglia in the LS (Fig. S1I, J). DEG analysis (Fig. S1K-M) further revealed dysregulated expression of 14-3-3 protein family members (*Ywhaq*, *Ywhag*, *Ywhaz*), indicating impairments in apoptosis regulation, stress response, and cell cycle control^36^.

### Different immune and genetic backgrounds altered neuronal composition and gene expression in the LS

**Figure 3:**
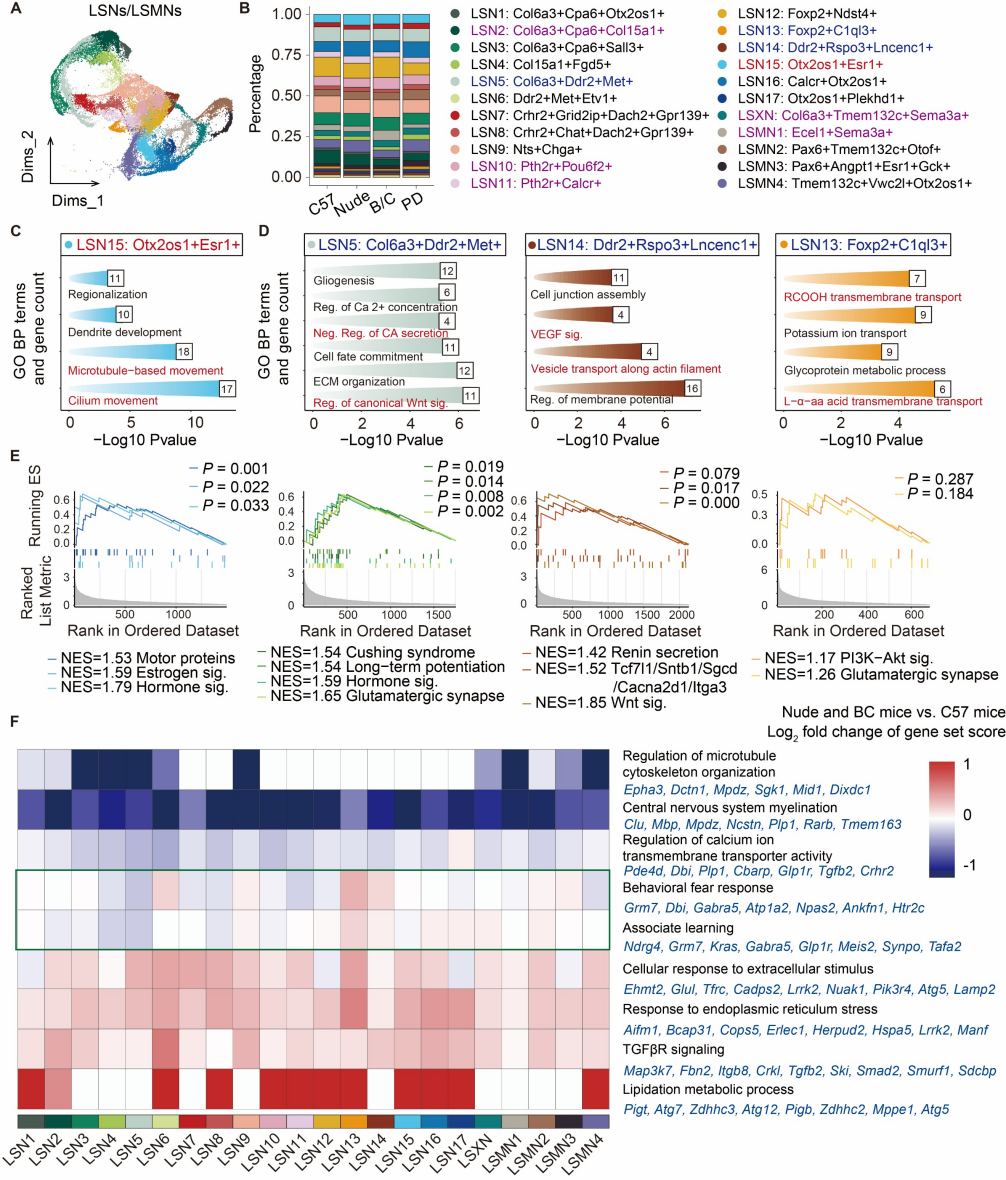
** Different immune and genetic backgrounds show the functional heterogeneity of neuronal subtypes in the LS. (A)** UMAP plot showing 22 neuronal subclusters with distinct transcriptome patterns. **(B)** Proportions of 22 neuronal subclusters within each cohort. **(C, D)** GO enrichment analysis of interested LSNs. Compared with C57J mice, LSN15 was upregulated both in nude and BALB/c mice **(C)**, while LSN5, 13, 14 were downregulated both in nude and BALB/c mice **(D)**. **(E)** GSEA analysis of the corresponding LSNs mentioned above. **(F)** The heatmap shows the log2 fold change (log2FC) of scores of interested gene set in 22 LS associated neurons, comparing nude and BALB/c mice to C57J mice. Representative genes of each interested gene set are indicated in blue.

The expression patterns of *Trpc4*, *Elavl2*, and *Rarb* were used to distinguish and annotate LS associated neurons, medial septal (MS) neurons, and striatal (STR) neurons, respectively (Fig. S2A-D). Subsequently, LS associated neurons were isolated for detailed analyses and classified into distinct subclusters based on characteristic gene signatures (Fig. 3A; Fig. S2E). This approach yielded 17 LS neuronal subclusters (LSNs), 1 LS complex neuronal subcluster (LSXN), and 4 neuronal subclusters localized at the interface between the LS and adjacent brain regions (LSMN). Proportional analysis revealed that LSN15 demonstrated significantly higher ratios in both nude mice and BALB/c mice, whereas LSN5, LSN13, and LSN14 showed markedly reduced abundance when compared to C57J controls from both same-batch sequencing cohorts as well as public dataset (Fig. 3B; Fig. S3A, B). Furthermore, specific neuronal subclusters displayed mouse strain-specific alterations, observed exclusively in either nude mice or BALB/c mice (Fig. S3C). LSN2 showed a reduced proportion in BALB/c mice; LSN10, LSMN1, and LSXN exhibited increased proportions in BALB/c mice, whereas LSXN showed the opposite trend, i.e., a decreased proportion in nude mice; LSN11 was selectively enriched in nude mice (Fig. S3C).

To elucidate the potential functional specificity underlying these characteristic alterations, Gene Ontology (GO) enrichment analysis was performed on marker gene sets of the aforementioned subclusters, and enrichment significance was then evaluated across all annotated subclusters (Fig. 3C, D; Fig. S3D). The GO terms marked in red visualize specific functional modules related to subclusters. More specifically, GSEA was conducted on four subclusters (LSN15, LSN5, LSN14, LSN13) to delineate their unique functional gene modules (Fig. 3E). The combinatorial analyses revealed that LSN15 exhibits heightened ciliary activity and elevated expression of multiple hormone receptors (including estrogen receptors), suggesting enhanced environmental sensing capacity and potential rapid responsiveness to peripheral signals. Conversely, subclusters with reduced proportions in nude and BALB/c mice (LSN5, LSN14, LSN13) showed characteristic enrichment in canonical Wnt signaling pathways, catecholamine secretion, renin secretion, long-term potentiation and glutamatergic synapse, indicative of potential axonal and synaptic transmission-related abnormalities.

Differentially represented LSNs specific in either nude mice or BALB/c mice also displayed unique pathway enrichments (Fig. S3D). For example, LSN10, LSMN1, and LSN2 were associated with memory formation and extinction, involving GO terms such as “Dendritic spine development”, “Associative learning”, “Visual learning”, and “Learning or memory”. This suggests potentially disrupted memory processes in BALB/c mice^37^. LSXN was enriched in the non-canonical “Wnt signaling pathway” (e.g., *Fgf2* and *Ror1*), and may also be associated with learning and memory behaviors^38^,^39^. Of note, existing literature has reported that BALB/c mice exhibit slower learning performance but more memory consolidation^40^, while nude mice perform worse than BALB/c mice in both learning and memory maintenance^41^. Among these findings, non-specific impairments in glia, axonal and synaptic transmission may be associated with learning acquisition deficits. Meanwhile, enhanced memory consolidation in BALB/c mice may be attributed to their potentially more stable dendritic spines and synaptic structures. Additionally, LSN11 was enriched in “Negative regulation of transmembrane receptor protein serine/threonine kinase (Neg. Reg. of tRS/T-K) signaling pathway”, suggesting neural pulse sensitization^42^. The marker genes of LSN11 including *Pth2r*, *Calcr*, *Acvr1*, were reported to be associated with pain response regulation^43^-^45^, suggesting that nude mice may have pain sensitization^46^.

Subsequent analysis of total neuronal DEGs and protein-protein interaction (PPI) enrichment revealed that nude and BALB/c mice also exhibited some common transcriptomic changes relative to C57J mice (Fig. S4A-C). The direct comparison between BALB/c mice and nude mice (data not shown) is primarily attributed to T-cell deficiency, which has been extensively discussed in previous studies. Therefore, it is not a major focus in the current research. Co-downregulated genes (Fig. S4B) were associated with axonal extension and transport, as well as clearance of toxic/harmful compounds, whereas co-upregulated genes (Fig. S4C) showed significant enrichment in voltage-gated potassium channel activity and Ras signaling pathway. Notably, enhanced potassium channel signaling has been reported to correlate with heightened anxiety phenotypes^47^. *Ndn* can inhibit the Ras signaling pathway, but its overexpression suppresses synaptic pruning, increases neuronal excitability, and also contributes to the promotion of anxiety^48^. Despite conducting sequencing analysis on pooled tissue samples from three groups, inter-group variability was specifically assessed through technical validation. Independent quantitative reverse transcription polymerase chain reaction (qRT-PCR) verification of critical gene expression changes across additional biological replicates consistently corroborated transcriptomic findings (Fig. S4D). Given the pronounced cellular heterogeneity inherent to neuronal populations, subcluster-specific DEG analyses were further performed (Fig. S4E). To achieve high-resolution visualization of functional perturbations, biologically meaningful DEG sets were selected and subsequently applied signature scoring to quantify their expression patterns (Fig. 3F). Specifically, a widespread reduction in neuronal myelination-related gene expression was also observed in all neuronal subtypes, which aligned well with the aforementioned glial-cell changes, and further supported the notion that in both immune compromised animals, myelination deficits may be present, as previously reported^30^-^33^. It is worth mentioning that although myelination related genes are robustly expressed almost exclusively in oligodendrocyte lineage cells, our data showed extremely low levels of these transcript expressions in neurons, yet with significant intergroup differences. Microtubule assembly and calcium ion transport exhibited significant downregulation in some LSNs. Conversely, environmental stress response and TGFβ receptor signaling pathway activities demonstrated global enhancement. The results suggest that nude mice and BALB/c mice may exhibit enhanced stress responses or sensitization of neural signaling excitability^49^,^50^.

Intriguingly, perturbations in fear behavior regulation pathways were detected (Fig. 3F). Representative genes of the pathways (“Behavioral fear response” and “Associate learning”) were further studied and verified. *Grm7*, *Glp1r*, and *Ndrg4* displayed consistent downregulation in both nude and BALB/c mice (Fig. S4F), suggesting impaired fear memory and associative learning^51^-^53^. *Grm7* in particular was also reported to be involved in neurodevelopment and myelination^54^. *Synpo* has been shown to be upregulated by the Th2 classic factor IL13^55^, and the results showed that it was only upregulated in BALB/c mice (Fig. S4G), indicating a potential enhancement of memory or fear memory maintenance^56^,^57^. *Tafa2*, associated with memory maintenance^58^, exhibited a more nuanced regulatory pattern, showing subtype- and strain-specific expression (Fig. S4H). For instance, it was upregulated in LSMN1 of BALB/c mice compared to other strains, though there was no statistically significant difference when considering all LSNs as a whole. In summary, while both BALB/c mice and nude mice may exhibit impaired memory acquisition, BALB/c mice may show rigidity in emotional memory (such as fear memory).

### Three-dimensional neuronal heterogeneity in the LS

**Figure 4:**
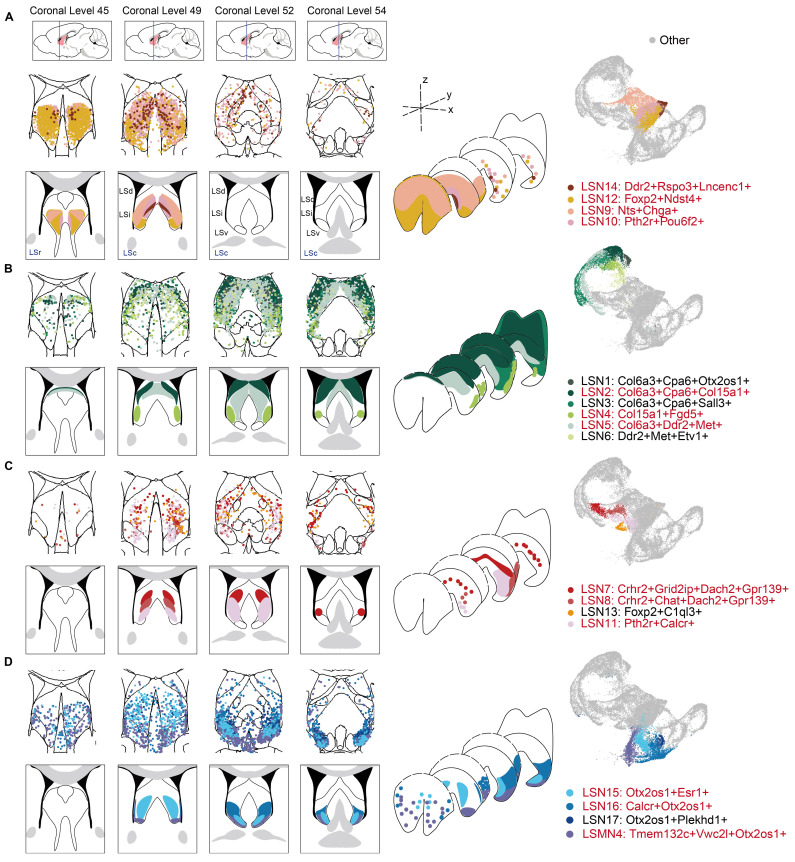
** Three-dimensional neuronal heterogeneity in the LS.** Spatial plot showing the distribution of LSNs in the MERFISH dataset across 4 representative coronal sections along the anterior-posterior axis: +0.945 mm (coronal level 45), +0.545 mm (coronal level 49), +0.245 mm (coronal level 52), +0.02 mm (coronal level 54). **(A)** From left to right, LSNs concentrated within rostral LS were displayed in 4 coronal sections, 3D structures, and UMAP plot, respectively. **(B)** shows the LSNs distributed within caudal-dorsal LS, **(C)** shows the LSNs distributed within caudal-intermediate LS, and **(D)** shows the LSNs distributed within caudal-ventral LS.

To systematically analyze three-dimensional neuronal distribution patterns, coronal serial sections were spaced and progressed sequentially along the rostral-caudal axis. Integrating multiplexed error-robust fluorescence *in situ* hybridization (MERFISH) data of the entire LS^59^, the defined LSNs as described above were systematically mapped and classified into four distinct categories based on their three-dimensional spatial distribution signatures (Fig. 4A-D). Incidentally, the spatial segregation patterns were also represented by their relative positioning in UMAP, suggesting a robust structure-gene expression (function-relevant) correspondence.

LSNs predominantly localized within the rostral LS (LSr) were first observed, comprised LSN9, LSN10, LSN12 and LSN14 (Fig. 4A; Fig. S5A). LSN12 was widely distributed on LSr, extending caudally into intermediate and ventral domains. LSN9 occupied a rostral-dorsal niche, transitioning caudally to an intermediolateral distribution, while LSN10 and LSN14 maintained medial positioning. LSN1-6 exhibited predominant dorsal localization with sparse rostral distribution (Fig. 4B; Fig. S5B). Notably, LSN2, LSN4, and LSN5 demonstrated well-circumscribed spatial boundaries. LSN2 was a particularly high-density subcluster in the caudal dorsolateral quadrant, whereas LSN5 concentrated within the caudal dorsomedial region and LSN4 occupied an intermediolateral position. Neuronal subclusters localized within the central LS, including LSN7, LSN8, LSN11, and LSN13, were categorized as a spatially distinct class. This group exhibited sparse representation in both rostral and caudal regions (Fig. 4C; Fig. S6A). Notably, LSN8 and LSN11 shared similar distribution profiles, occupying ventral-biased positions. LSN7, defined by *Crhr2* expression without *Chat*, displayed a dorsal-shifted distribution, demarcating the boundary between the dorsal LS (LSd) and intermediate LS (LSi). Lastly, a category comprising ventral and paraventral LSNs was delineated, including LSN15, LSN16, LSN17, and LSMN4. This category also exhibited exceptionally sparse rostral representation (Fig. 4D; Fig. S6B). In addition, there are also some LS-associated neuron distributions at the junction of LS and adjacent brain regions (Fig. S6C).

Concurrently, the feasibility of employing single-gene markers for precise spatial localization was evaluated, with candidate genes predominantly derived from the established marker gene sets of the aforementioned LSNs. *Foxp2* served as an effective marker for rostral-localized LSNs. Within the rostrointermediate domains, *Nts* exhibited preferential localization to lateral subregions, whereas *Pou6f2* demarcated medial subdivisions (Fig. S7A-C). While somatostatin (*Sst*) has been extensively studied in the LS and is conventionally associated with dorsal distributions^60^,^61^, the findings here revealed that multiple LSNs expressed *Sst* with insufficient spatial specificity. Notably, a rostral co-expression niche where *Sst* and *Nts* colocalized was observed, challenging the traditional dorsally-restricted *Sst* paradigm. *Col6a3* and *Cpa6* covered the LSd and LSi. Similar to the distribution characteristics of LSN2, *Col15a1* marked the dorsolateral distribution; Similar to the distribution characteristics of LSN5, *Met* and *Ddr2* marked the dorsomedial side (Fig. S7D-F). The LSNs defined by *Crhr2*, *Dach2*, and *Grid2ip* were highly overlapping, and their characteristics in *in situ* hybridization (ISH) results from the Allen Brain Atlas were also quite similar, all defining medial distribution. *Chat*-positive neurons were rare in the LS and distributed on the ventral side of the LSi (Fig. S8A-C). Along the caudoventral axis, *Otx2/Otx2os1* covered multiple LSNs, with *Esr1* and *Calcr* more concentrated on the ventral side of the LSi (Fig. S8D-F).

Overall, the only neuronal subcluster that gained significant enrichment in both nude and BALB/c mice was LSN15, characterized by *Otx2os1* and *Esr1 expression*, exhibiting a caudoventral distribution. Additionally, the intermediate-ventrally located LSN11, which expresses *Calcr*, was elevated only in nude mice. Conversely, neuronal subclusters LSN5, LSN13, LSN14 (mainly marked by *Met* or *Ddr2*) were reduced in both nude and BALB/c mice and localized within medial domains regardless of rostrocaudal position. However, LSN10, LSMN1, and LSXN, which were also located within the medial LS, showed specifically increased expression in BALB/c mice (mainly marked by *Pou6f2* or *Sema3a*). Downregulated only in BALB/c mice, LSN2 co-expresses *Col6a3*, *Cpa6*, and *Col15a1* and is strictly localized in the dorsolateral region of the LS (Fig. 4; Fig. S5-8). In other words, the dorsal, medial, and ventral regions of the LS may be more susceptible to peripheral immune influences. Given the identical strain background between nude and BALB/c mice, the T-cell deficiency led to increased reactivity in the ventromedial LSN11 and dorsal LSN2, while the medially-located LSN10, LSMN1, and LSXN showed reduced percentages. Combined with functional annotation results, these findings suggest that emotional memory may also be modulated by peripheral immune bias.

To assess the evolutionary conservation of LSNs, publicly available human snRNA-seq dataset^5^ derived from the LS was subject to similar analyses (Fig. S9A). Among the human LSNs, *COL6A3* expression was negligible, while *CPA6* exhibited higher cross-species conservation. Combinatorial expression patterns of *COL15A1*, *MET*, and *FGD5* enabled further stratification into three subclusters that showed marked similarity to mouse caudodorsal LSNs (Fig. S9B). Interestingly, mouse caudoventral LSNs characterized by *Esr1* and *Calcr* also demonstrated substantial cross-species conservation (Fig. S9B). Unexpectedly, human LS samples displayed nearly undetectable levels of *NTS* and *CHAT* transcripts (Fig. S9B).

### Spatial-specific subregional projection networks in the LS highlighted *Esr1*-positive neurons as potential therapeutic targets for anxiety

**Figure 5:**
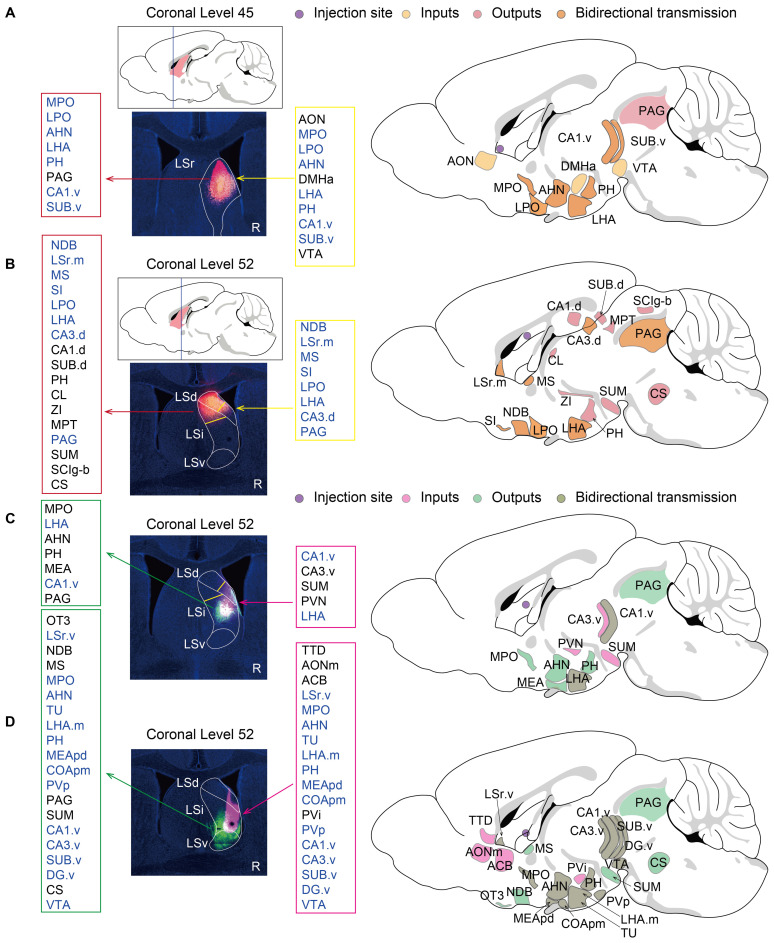
** Spatial-specific subregional projection networks in the LS.** The left panels represent the actual injection sites, while the right side provides a schematic diagram of injection sites and projection network. The framed annotations on both sides of the injection sites specify the input and output projections of the respective LS subregion (R-label means the right hemisphere of the mouse brain). **(A)** The Bregma level is +0.7 mm, and the injection site is within rostral LS. **(B-D)** The Bregma level is +0.1 mm. The injection site of **(B)** is within caudal-dorsal LS, **(C)** is within caudal-intermediate LS, and **(D)** is within caudal-ventral LS.

To dissect the functional implications of the spatial topography encompassing heterogeneous LSNs, comprehensive neuroanatomical tracing was performed to map region-specific projection networks. Firstly, a section containing LSr (AP: +0.7 mm, ML: +0.5 mm, DV: -3.5 mm) was selected whose projection network may mainly involve LSN9 (*Nts*-positive), LSN12 (*Foxp2*-positive) and *Sst*-positive LSNs (Fig. 4A, 5A; Fig. S7A-C, S10A). A significant bidirectional projection relationship was found between this subregion and the ventral CA1 (CA1.v) as well as the hypothalamus. In addition, this subregion received projections from the anterior olfactory nucleus (AON), which were related to olfactory induced social and defensive behaviors^62^. This subregion also possessed projections to the periaqueductal gray (PAG) which is related to anxiety, depression, fear learning, defense, and pain regulation^63^,^64^(Fig. 5A; S10A).

Further, a caudal section was selected to investigate the projection networks of the LSd, LSi, and LSv, respectively. In terms of the LSd (AP: +0.1 mm, ML: +0.35 mm, DV: -2.3 mm; Fig. 5B; S10B), the dorsal subregion was spatially demarcated encompassing both the LSd and LSi, rather than the conventional entire LSd, due to the evident projection heterogeneity between the dorsal and ventral compartments of the LSd^65^. The neuronal composition in this subregion consisted of LSN2 (*Cpa6-* and *Col15a1*-positive), LSN3 (*Cpa6-* and *Sall3*-positive), LSN5 (*Ddr2-* and *Met*-positive), and *Sst*-positive LSNs (Fig. 4B; Fig. S7D-F). In addition to bidirectional projections from the hypothalamus and dorsal hippocampus, this subregion also projected to the thalamus and midbrain. Neural signaling from the dorsal CA3 (CA3.d) is known to augment fear-related defensive behaviors in mice^65^.

The projection network within the mid-LS, encompassing the ventral compartment of the LSd and the central subdivision of the LSi (AP: +0.25 mm, ML: +0.5 mm, DV: -3.4 mm; Fig. 5C; S10C) was investigated. This subregion predominantly comprised neuronal subclusters LSN7, LSN2, LSN3, and LSN5 (Fig. 4B, C), which receive ventral hippocampal inputs originating from the CA1.v and ventral CA3 (CA3.v). Notably, CA3.v has been documented to project to the ventrolateral LSd, a pathway implicated in suppressing fear-related freezing behavior^65^. Therefore, LSN2 and LSN5, distinguished by their divergent spatial topography and heterogeneity in projection circuitry, likely engage in fear modulation through dissociable mechanisms. Furthermore, LSN7 (expressing *Crhr2*) has been reported to promote anxiety-like behaviors via its projections to the anterior hypothalamic nucleus (AHN)^13^.

**Figure 6:**
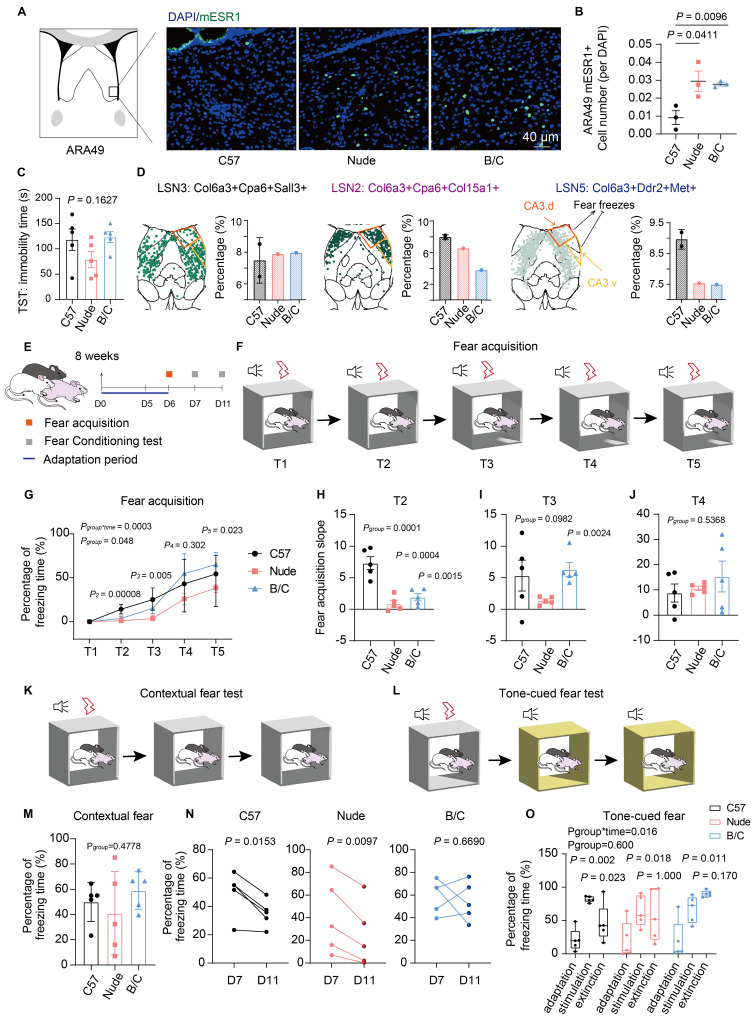
** BALB/c mice exhibited enhanced fear memory maintenance. (A)** ESR1 immunofluorescence imaging of the magnified ventral LS. **(B)** Quantitative analysis of the ESR1-positive cell counts per DAPI among C57J, nude and BALB/c mice (mean ± SEM, n = 3, Student's t test was used). **(C)** Statistical analysis of the duration of immobility in the TST among mouse strains (mean ± SEM, n = 5, one-way ANOVA was used). **(D)** Spatial localization of LSN2, 3, 5 in one representative section and the ratio difference across strains. The orange framework represents the projection of CA3.d, which participates in fear enhancement, while the yellow framework represents the projection of CA3.v, which participates in fear suppression. **(E)** Schematic diagram showing the experimental procedures of fear behavioral test. **(F)** Schematic diagram of fear acquisition. **(G)** Line chart showing the changes in freezing time ratio of different mouse strains with fear training (mean ± SEM, n = 5, repeated measure ANOVA with Bonferroni posttest and sample effect analysis were used). **(H-J)** Statistical analysis of the slopes of fear acquisition of three mouse strains at the T2, T3, and T4 stages (mean ± SEM, n = 5, one-way ANOVA and Student's t test were used). **(K)** Schematic diagram of contextual fear test. **(L)** Schematic diagram of tone-cued fear test. **(M)** Statistical analysis of the contextual fear test on D7 (mean ± SEM, n = 5, one-way ANOVA was used). **(N)** Statistical analysis of the contextual fear extinction of three mouse strains during the period from D7 to D11 (Scatter plot, n = 5, paired t test was employed). **(O)** Tone-cued fear and fear extinction of three mouse strains on D7 were illustrated by boxplot (Median ± upper and lower quartiles, n = 5, repeated measure ANOVA with Bonferroni posttest and sample effect analysis were used).

Along the Z-axis of the caudal LS (LSc), potential projection circuits within the ventral and ventral-adjacent subregions were further assessed (AP: +0.1 mm, ML: +0.5 mm, DV: -4.0 mm; Fig. 5D; S10D). This compartment exhibits marked heterogeneity in anxiety regulation^6^, likely mediated by divergent projections engaging distinct LSNs, given the extensive spatial overlap of neuronal subpopulations. Notably, the findings here revealed that LSN15 (*Esr1*-positive) exhibited an intermediate-biased distribution within the LSr and proximity LSr, transitioning to a ventral concentration in the LSc (Fig. 4D; Fig. S6B, S8D-F). LSv-derived *Esr1*-positive neurons, previously associated with addiction via projections to the ventral tegmental area (VTA)^10^, may share overlapping neurocircuitry mechanisms underlying both addiction and anxiety phenotypes^66^-^68^. Also, ESR1, an estrogen receptor, has been reported to be associated with anxiety^69^-^71^. Therefore, ESR1 protein levels in the LS were examined and the expression pattern was consistent with RNA localization (Fig. 6A). Both BALB/c and nude mice showed significantly higher expression compared wildtype C57J mice (Fig. 6A, B). Therefore, we speculate that either T cell deficiency or type 2-biased T cell responses may enhance ESR1 expression, ESR1 neurogenesis, and/or protection, thereby promoting anxiety. Meanwhile, there were no statistically significant differences in depressive behavior among strains (Fig. 6C). The impact on depressive responses requires more detailed and thorough investigations. Additionally, projections between the ventral or ventral-adjacent subregions of the LS and the medial amygdala (MEA) were identified, though their functional significance remains to be elucidated (Fig. 5C, D; S10C, D).

### BALB/c mice exhibited enhanced fear memory maintenance

Dorsal LSN2 and medial LSN10, LSMN1, LSXN appeared to be involved in memory acquisition, consolidation, or extinction (Fig. S3D). DEG analyses of LSNs revealed enrichment in fear response and associative learning pathways (Fig. 3F; S4F-H). These findings suggest that nude and BALB/c mice may exhibit perturbations in emotional memory. Among them, LSN2, which was downregulated exclusively in BALB/c mice, was localized in the caudal dorsolateral LS and identified as one of the *Sst*-positive LSNs (Fig. S7C). These *Sst*-positive neurons have been previously reported to inhibit fear and promote fear extinction^14^,^72^. Additionally, LSN2 co-expresses genes include *Cnr1*, *Glp1r* and *Ptchd1*, which have been implicated in memory extinction^73^-^75^. Meanwhile, LSN3 and LSN5 subpopulations were identified, which occupy similar spatial positions but did not express *Sst* (Fig. 6D; S7C). Among them, LSN5 was located medially and exhibited spatial overlap with parvalbumin (PV)-positive neurons^65^, which likewise lack *Sst* expression and are known to play a key role in fear acquisition^14^. Notably, LSN5, which was downregulated both in nude and BALB/c mice, was also marked by genes such as *Ddr2* and *Met*, which have also been implicated in fear acquisition^76^. Since the dorsolateral LS has been reported to exhibit functional heterogeneity in fear modulation^6^,^65^ (Fig. 6D), fear response differences in mice with different immune backgrounds were further investigated. During fear acquisition, both BALB/c and nude mice showed delayed fear encoding, yet BALB/c mice displayed significantly higher freezing rates (Fig. 6E-J). No significant differences were observed in contextual or tone-cued fear tests across strains, whereas BALB/c mice exhibited pronounced fear extinction deficits over time (Fig. 6K-O). Given that nude and BALB/c mice share similar phenotypes of high anxiety-like behaviors (likely associated with reduced myelination, astrocytic dysfunction, and increased *Esr1*-positive LSNs), these findings suggest a potential dissociation between heightened anxiety and delayed fear extinction, the latter which might be linked to LSN2. Meanwhile, LSN10, LSMN1, and LSXN subpopulations may also play similar roles, potentially contributing to memory consolidation.

## Discussion

The lateral septum (LS) exhibits evolutionarily conserved mechanisms in regulating emotional circuitry and has specialized in primates to form more complex social adaptation modules, which could be critical for species survival. However, the neuronal composition, molecular signatures, and three-dimensional spatial topography of LSNs remain poorly understood. In this study, 22 LS associated neurons were identified using snRNA-seq and integrated MERFISH and ISH data to spatially map all neurons within a three-dimensional LS atlas. It is rather striking that overall, neurons that are anatomically colocalized tend to share similar transcriptomic features as indicated by adjacent positions on the UMAP plot. It remains to be investigated their relationships during development. Functional annotations and predictions were further established by correlating neuronal transcriptomic profiles, projection patterns, and behavioral analyses in three mouse strains including wildtype C57J, and two immune abnormal strains, nude and BALB/c (both originate from the same genetic background). Given the prominent circulatory immune dys-regulation observed in LS dysfunction-associated psychiatric disorders, neuronal and non-neuronal alterations in the LS were comparatively analyzed across the aforementioned mouse strains. This work highlights potential interactions between LS function and peripheral as well as central immune status in the context of mood regulations and fear learning (Fig. S12).

The functional heterogeneity of the LS under physiological conditions arises from its complex topographic-projection architecture and overlapping boundaries between neuronal subpopulations. However, environmental factors can also influence LSN characteristics to alter corresponding functions. Beyond local microenvironmental influences, accumulating evidence suggests that peripheral circulatory factors, including immune and hormonal status, participate in central neural circuit regulation, modulating behaviors such as anxiety, fear, social interaction, and cognition^69^,^71^,^77^-^80^. Nonspecific depletion of T cells has been shown to impair central nervous system (CNS) development by reducing synaptic plasticity and myelination, leading to anxiety and cognitive dysfunction^41^,^81^,^82^. Intriguingly, CD4-T cell reconstitution rescues these phenotypes, whereas CD8-T cells do not^20^. Paradoxically, both BALB/c mice (a high-anxiety model) and anxiety patients with 22q11.2 deletion syndrome exhibit elevated CD4+/CD8+ ratios^27^, suggesting that helper T cells, primarily functioning through cytokine secretion, play a critical role in emotional regulation, with outcomes influenced by cytokine secretion biases. Notably, Th2-skewed immunity does not always correlate with emotional disorders: while Th2 dominance characterizes diseases like atopic dermatitis and asthma, only a subset of these patients develops trait anxiety, which further correlates with elevated Th2-related cytokine levels^26^. Interestingly, IL-4 and IL-13, classic type 2 cytokines, are generally observed to exert neuroprotective effects in the adult CNS. However, enhanced Th2 reactivity during developmental stages can lead to CNS developmental abnormalities (including demyelination)^30^. These findings indicate that simplistic Th1/Th2 polarization cannot fully explain the complexity of immune-neural crosstalk. Future studies should distinguish between developmental and trait effects. Based on the observations in this study, BALB/c mice with CD4-T cell predominance and Th2-skewed response displayed heightened anxiety, while CD4-/CD8-T cell-deficient nude mice also exhibited similar high-anxiety phenotypes. Subsequent investigations revealed that both BALB/c mice and nude mice shared pathological features of myelination deficits and astrocyte dysfunction—both proven to be anxiogenic mechanisms. This suggests that either the absence of T cells or their polarized response patterns (e.g., Th2 bias) may disrupt CNS glial support systems via distinguished mechanisms^30^,^83^, ultimately manifesting as overlapping anxiety-related neuropathology.

ESR1 (also known as estrogen receptor α) -positive neurons are predominantly distributed in the BNSTpr (Bed nuclei of the stria terminalis, posterior division, principal nucleus), MEA (Medial amygdala), MPO (Medial preoptic area) and VMHvl (Ventromedial hypothalamic nucleus, ventrolateral part), with moderate expression levels observed in the AHN, LSv, and PAG^84^. While these neurons exhibit distinct functional properties across different brain regions, they share a common characteristic of being highly responsive to peripheral environmental changes^85^. In this study, limited *Esr1* expression was observed in the LSv or adjacent LSv regions, encompassing LSN15 and LSMN3 subpopulations. The proportion of LSN15 was significantly increased in both nude mice and BALB/c mice. Gene expression enrichment analyses suggested that LSN15 might exhibit robust ciliary activity and hormone receptor expression, establishing a biological foundation for heightened environmental responsiveness, as previously reported^85^. In terms of function, *Esr1*-positive neurons exhibit a high degree of heterogeneity. *Esr1*-positive neurons in the MPO promote maternal behaviors, whereas those in the BNSTpr suppress such behaviors^86^. Notably, *Esr1*-positive neurons in most brain regions show increased activity during aggressive behaviors (regardless of gender), with the LSv being a notable exception^84^. Consequently, *Esr1*-positive neurons in the LSv are considered to be involved in inducing and maintaining aversive responses to stimuli^87^. A recent study demonstrated that *Esr1*-positive neurons in the LSv contribute to addiction-like behaviors^10^. Given addiction and anxiety often co-occur and are regulated by shared neural circuits^88^, *Esr1*-positive neurons may also play an anxiety-provoking role, including excessive aversion to open spaces and light stimuli. Although no direct evidence currently establishes *Esr1*-positive LSNs as being directly involved in anxiety behaviors, several lines of evidence support their potential role (regardless of gender): 1) elevated *Esr1* expression has been observed in anxiety-prone mice^70^,^89^, 2) specific activation of *Esr1* has been shown to induce anxiety-like behaviors^90^, 3) genome-wide association studies (GWAS) of human anxiety disorders and post-traumatic stress disorder (PTSD) cohorts have also identified disease-linked *ESR1* variants^71^,^78^,^91^. Taken together, we reasonably speculate that *Esr1*-positive LSNs are likely implicated in promoting anxiety. To investigate impact of peripheral immune regulation on LSN15, further studies will examine *Esr1* activation patterns and behavioral outcomes in an ovalbumin (OVA)-induced murine model, which is characterized by enhanced Th2 reactivity and anxiety-like phenotypes^92^,^93^.

Regarding depressive phenotypes, GABAergic *Esr1*-positive neurons in the MPO have been shown to exert antidepressant effects^94^. However, no statistically significant differences were observed among the three mouse strains. Of note, considering that *Esr1* functions may vary significantly depending on projection patterns and brain regions, it remains challenging to definitively determine which specific neuronal subpopulations contribute to depression. Additionally, *Esr1* exhibits pronounced brain region-specific sexual dimorphism, yet it is consistently involved in the regulation of multiple key emotional behaviors^71^,^84^,^95^. The observed phenomena and derived interpretations in this study are mainly applicable to male mice; thus, extrapolation to female mice requires additional considerations.

It is noteworthy that, in addition to potential direct immune factors, mice from a BALB/c background (including nude mice) exhibit other pro-anxiety mechanisms. Compared to C57J mice, BALB/c mice show higher levels of corticosterone, leading to dysregulation of the hypothalamic-pituitary-adrenal (HPA) axis negative feedback and promoting anxiety^96^. Similarly, dysregulation of glutamatergic signaling and BDNF modulation is also a significant contributor to anxiety in BALB/c mice^97^, which may be indirectly influenced by gut-immune interactions^98^. Consequently, BALB/c mice inherently possess a higher baseline stress sensitivity^99^. Under T cell deficiency, nude mice exhibited impaired myelination and synaptic pruning^83^,^100^, which may involve the superimposed effects of the aforementioned pathways, thereby maintaining or exacerbating strain-related anxiety phenotypes. However, nude mice display lower HPA axis reactivity^101^, in contrast to BALB/c mice, which theoretically should reduce anxiety—yet this is contradicted by actual behavioral observations. These findings also suggest that corticosterone levels and the HPA axis may be indirectly or directly affected by immune disturbances. Therefore, the mechanism leading to anxiety is likely the result of multiple interacting factors.

In traditional studies, neuropeptides serve as functional markers for neuronal classification. SST-positive neurons are typically described in the LSd and recognized as key inhibitory neuropeptide markers in the LS, often expressed in GABAergic neurons^60^. However, this current study revealed a broader distribution of *Sst*-positive neurons, potentially spanning subpopulations LSN2, 6, 9, 10, and 12. These mainly reside in the LSr and dorsal LSc, with sparse distribution in the LSi and LSv. Given the significant divergence in their transcriptomic signatures, we hypothesize that *Sst*-positive neurons in distinct spatial domains may exert divergent or even opposing functions. CA3.d projections target PV-positive neurons in the head of dorsal LSc, enhancing fear responses, whereas CA3.v projections innervate SST-positive neurons in the tail of dorsal LSc, suppressing fear^65^. In this study, LSN2, which received CA3.v inputs, was found markedly reduced in BALB/c mice, thereby disinhibiting fear. Since PV and SST expression rarely overlap, the sparse PV-positive neurons in the dorsal-medial of the LSd likely originate from LSN3 and LSN5. These neurons promote fear, yet are both reduced in both nude mice and BALB/c mice, underlying their heterogeneous fear-test responses. Additionally, *Sst*-positive neurons in LSr or adjacent subregions mainly derive from LSN6 (*Ddr2*, *Met* and *Etv1* positive), LSN9 (*Nts* and *Chga* positive), LSN10 (*Pth2r* and *Pou6f2* positive) and ​LSN12 (*Foxp2* and *Ndst4* positive) subpopulations, which warrant more nuanced investigation for functional annotation.

While the projections and functional studies of the medial LSi remain limited, the highly heterogeneous neuronal composition within this subregion underscores its potential significance. Neuronal clusters ​LSXN, LSMN1, LSN10, and LSN14 are densely localized in the medial LSi near LSr. Notably, ​LSMN1 and LSN10 are ​elevated exclusively in BALB/c mice, suggesting their potential involvement in ​peripheral immune-neural crosstalk. The medial LSi is predominantly populated by ​LSN5 and LSN6, which, as previously described, may participate in ​fear regulation. The spatial segregation of these clusters within medial LSi highlights a potential subregional role as another ​functional hub integrating environmental cues (e.g., immune signals) with emotional behaviors. Future studies should delineate how LSi subdomains coordinate these processes through distinct projection targets (e.g., hypothalamus, amygdala) and molecular pathways.

Here, we would like to emphasize that the major value of this study is the construction of a three-dimensional (3D) neuronal atlas of the entire LS in not only wildtype C57J mice but also two genetically-linked mice with different immune abnormalities, i.e. BALB/c (Th2-skewed immunity) and nude (T cell deficient) mice. Nude mice in this study are *Foxn1* knockout descendants from the BALB/c background. Therefore, even correlative findings between the two strains with abnormal immunity could stem from one gene (i.e., *Foxn1*). The behavioral phenotype and LS neuronal atlas changes as well as non-neuronal cell changes are quite robust and have enabled correlative results to be drawn. Interestingly, such high-throughput data processing has generated correlative suggestions with actual predictive power, e.g., the fear-learning, memory, and extinction phenotypes that were suggested from the transcriptomic data and behaviorally validated. This 3D-neuronal atlas establishes a framework for future mechanistic and causal studies aimed at developing prospective therapeutic strategies for emotional disorders via peripheral interventions.

Nevertheless, this study still has several limitations. The identification of subpopulations and DEG analysis in snRNA-seq depend on nuclear mRNA and its precursor expression, which may differ from cytoplasmic mRNA and protein levels. Thus future research should incorporate more specific causal studies.

## Materials and Methods

### Animals

All animal experiments and procedures were approved by the Institutional Animal Care and Use Committee (TJAA06621110) of Tongji University, Shanghai, China. This present study involves male mice of three different genetic backgrounds, including C57BL/6JNifdc, BALB/c, and BALB/c nude. They were purchased from Charles River Laboratories (Beijing) at 8 weeks of age. The mice were housed in an SPF environment within a laboratory animal facility, which was maintained at temperatures between 21°C and 23°C and humidity levels of 40% to 60%, under a 12 h light/dark cycle, with access to food and water *ad libitum*.

### Estimation of sample size

This study was an exploratory study, therefore there was no need to estimate the sample size in advance to meet statistical efficiency. To ensure adequate nucleus yield and minimize batch effects due to individual variability, brain tissues from three mice of the same strain were pooled together and processed as one sample for snRNA-seq. To validate the reproducibility and stability of our experimental strategy, two independent snRNA-seq runs were performed using the C57BL/6J samples. These two independent C57BL/6J samples were generated: C57J-1 and C57J-2, each derived from a separate set of three mice. These datasets showed highly consistent transcriptomic profiles, confirming the robustness of the protocol. Nude and BALB/c samples (labeled as Nude and B/C, respectively) were also processed in parallel with C57J-2 using the same protocol. Publicly available C57J snRNA-seq dataset was also incorporated to enhance the accuracy of LSN identification and spatial localization. To further reduce the risk of false-positive or false-negative findings in the identification of DEGs due to individual-level outliers, mRNA- or protein-level validations were performed in an independent cohort of nine mice (three C57J, three nude, and three BALB/c mice). These animals were not used for sequencing but served as biological replicates to confirm key results.

### Tissue processing

Six C57BL/6J, three BALB/C and three nude male mice were deeply anesthetized via intraperitoneal injection of pentobarbital sodium at a concentration of 80 mg/kg, followed by perfusion with ice-cold 1× PBS to remove residual blood. Subsequently, for tissue isolation, the mouse brain was sagittally bisected along the midline, and the lateral septum (LS) tissues were micro dissected under a stereomicroscope on ice-cold PBS to minimize tissue degradation. Samples were flash-frozen in precooled isopentane and stored at -80°C for subsequent nuclei isolation.

### Single nucleus isolation and single-nucleus RNA sequencing

Nuclei were isolated from fresh-frozen tissue, according to the published protocol with the several modifications. Briefly, frozen tissue was Dounce homogenized with 10 strokes of a loose pestle followed by 10 strokes with a tight pestle at 4°C in a low sucrose buffer (0.32 M sucrose, 1 mM KH_2_PO_4_, 1 mM MgCl_2_, 0.2 mM EDTA, 0.1 mM DTT, 3 μM Actinomycin D and Protease/RNase inhibitor) with 0.25% Triton X-100 added. After homogenizing, they were filtered through a 40 μm strainer and mixed with equal volume of high sucrose buffer (2.2 M sucrose). The filtered and mixed crude nuclei preps were gently loaded in high sucrose buffer. Samples were centrifuged at 13,000 × g for 45 min at 4°C. Pellets were rehydrated in concentration gradient sucrose solution with 0.4 U/μL RNase Inhibitor, transferred in new Eppendorf tubes and centrifuged for 5 min at 1,200 × g at 4°C. Finally, purified nuclei were re-suspended in 0.4% BSA PBS Buffer with 0.4 U/μl RNase Inhibitor, stained with trypan blue and counted using cell counting plate. A total of 16,000 estimated nuclei from each sample were loaded on the 10X Single Cell work platform. Library construction was accomplished using the Chromium Single Cell 3' Library according to the manufacturer's guidelines. Finally, libraries were sequenced using the Illumina Novaseq 6000 System platform.

### Single-nucleus RNA sequencing data cleaning and processing

The sequenced reads were aligned to the mouse genome (mm10), using the Cellranger 7.0.1 software. Downstream analyses were mainly processed by the Seurat R package (5.0.0). To filter out low-quality cells, a standard pipeline was established: cells with 200 < features < 6,000, 300 < gene counts < 50,000, mitochondrial gene percentage < 2%, log_10_(nCount_RNA) / log_10_(nFeature_RNA) > 0.8 were retained and genes expressed in less than 3 cells were also removed (Fig. S11A-D). Subsequently, the doublet rate for each sample was estimated, with an average rate of approximately 10%, indicating acceptable data quality (Fig. S11E, F). To correct for batch effects, an integration strategy based on Robust Principal Component Analysis (RPCA) was applied, using the top 5,000 highly variable genes identified in each sample as anchor features. Prior to integration, predicted doublets were removed to ensure the accuracy of downstream analyses. To visualize clusters, results were generated using Uniform Manifold Approximation and Projection (UMAP) using the top 50 mutual nearest-neighbor corrected PCs. Cells were clustered using the FindClusters function in Seurat with resolution = 2. General cluster identity was determined using previously identified transcriptional markers of cell types, including neuron (*Snap25*), astrocyte (*Gja1*), oligodendrocyte lineage (*Mog*, *Pdgfra*), microglia (*Selplg*), ependymal (*Fam216b*), endothelial (*Flt1*), mural cell (*Rgs5*), and tanycytes (*Tiam2*).

### Marker genes characteristics of LSNs

Previously published^5^ markers were used to identify the neural clusters from the striatum (*Rarb*), medial septum (*Elavl2*), and LS (*Trpc4*). Once the LS associated neurons were extracted, the FindAll-Markers and Dimplot were applied to find the undefined neuronal subclusters. In order to describe the biological definition and function of the subclusters, marker genes of defined subclusters were computed by the FindAllMarkers. The top 200 marker genes from interesting subclusters were selected as the filter gene set for further functional enrichment analysis.

### Differentially expressed genes (DEGs) and Enrichment analysis

DEGs between C57J cohort and BALB/c, nude cohort were calculated by the FindMarkers with the Wilcoxon rank-sum test. Significant DEGs were defined with expression log2 fold change > 0.5 and adjusted *p-*value < 0.05. The number of DEGs across distinct neural subclusters was also calculated. Subsequently, the R package ClusterProfiler was employed to perform gene ontology (GO) and Kyoto Encyclopedia of Genes and Genomes (KEGG) enrichment analyses on specific gene list. The present terms with a *p-*value below 0.05 were defined significantly enriched. GSEA was performed using gseGO and gseKEGG functions in clusterProfiler, with pathways selected based on |NES| ≥ 1 and *p-*value < 0.05. Visualization was done using ggplot2. Single-cell expression matrices were aggregated into pseudobulk profiles by AverageExpression function in Seurat, followed by normalization via limma package. Finally, the heatmaps of the average expression levels were plotted for the DEGs by pheatmap package.

### Protein interaction network analysis

The upregulated or downregulated genes from different subclusters in nude and BALB/c mice were imported into the STRING database to construct the interconnections of the corresponding proteins. The node relationship data were imported into Cytoscape 3.10.3 software. Protein-protein interaction (PPI) networks were constructed using the degree index to determine the importance of nodes.

### Integration of MERFISH data with snRNA-seq data

To investigate the 3D spatial distribution of neuronal subpopulations, annotated snRNA-seq data were integrated with published MERFISH spatial datasets (500 gene panels)^59^. In brief, the anchor-based transfer method in the Seurat package was employed to migrate cell type annotations from snRNA-seq data to MERFISH data. This approach leverages the principle of canonical correlation analysis (CCA), identifying a shared low-dimensional space between multimodal datasets to enable cross-modality cell type prediction. Specifically, integration analyses were performed for distinct anatomical regions defined in the MERFISH dataset, focusing on cells annotated with the parcellation structure in the lateral septal nucleus caudal part, lateral septal nucleus rostral part, lateral septal nucleus ventral part, and medial septal nucleus. During data preprocessing, the MERFISH dataset underwent LogNormalize normalization to mitigate technical biases caused by variations in sequencing depth across cells. The FindTransferAnchors function was then applied to identify anchors between the reference dataset (snRNA-seq) and the query dataset (MERFISH). This process utilized CCA to co-embed both datasets into a low-dimensional feature space (the first 30 dimensions were selected in this study), capturing shared biological features across modalities. The k.anchor parameter was set at 5 to ensure anchor parameter prediction specificity and stability, while k.score = 30 was used to provide robust scoring of anchors. Following anchor determination, cell type annotation transfer was implemented via the TransferData function. This function performs weighted predictions for each MERFISH query cell in the CCA feature space based on identified anchors, enabling accurate label migration. During prediction, the k.weight parameter was set to 50, meaning that the cell type labels were determined by aggregating information from the 50 nearest-neighbor anchors, thereby enhancing annotation reliability.

### Immunofluorescence analysis

Tissue sections were rinsed three times (10 min each) in 1× PBS containing 0.1% Triton X-100 (T-PBS) followed by a 1 h incubation in blocking solution (containing 2% normal donkey serum, 0.1% Triton X-100, and 1% bovine serum albumin in 1× PBS) at room temperature (RT). And sections were then stained with rabbit anti-IBA1 (1:500, Wako), chicken anti-GFAP (1:2000, Invitrogen), rabbit anti-Estrogen Receptor-α (1:1500, Millipore) overnight at 4°C. Sections were rinsed three times (10 min each) with T-PBS after removing the primary antibodies, and then secondary antibodies donkey anti-rabbit Alexa Fluor 488/647 (1:1000, Jackson Immuno Research), and donkey anti-chicken Alexa Fluor 488 (1:1000, Jackson Immuno Research) were applied for 1 h at RT. Sections were rinsed two times (10 min each) in 1× PBS, and mounted in DAPI Fluoromount (SouthernBiotech). Staining sections were imaged using Zeiss LSM800 laser scanning confocal microscope.

### Open field test (OFT)

Prior to behavioral testing, all mice were allowed a 7-day acclimation period in the facility under standardized conditions (temperature: 22 ± 1°C; 12-h light/dark cycle; *ad libitum* access to food and water) to minimize stress from transportation and environmental novelty. An OFT was applied to assess anxiety-like behavior among mice with different genetic backgrounds. Mice were introduced to a square open-field arena (27.6 × 27.6 cm^2^) for 10 min to test ambulatory episodes total speed and ratio of ambulatory distance in the central area by Activity Monitor 7 ENV-256T software. Considering the sensitivity of BALB/c mice to alcohol, the chamber was cleaned with water instead of alcohol after each trial.

### Elevated plus maze test (EPM)

EPM was also used to assess degree of anxiety-like behavior. Prior to behavioral testing, all animals were acclimated to the testing room about 60 min to minimize stress-induced behavioral variability. The EPM apparatus consisted of two opposing open arms (30 × 5 cm, without walls) and two enclosed arms (30 × 5 × 15 cm, with opaque walls) elevated 50 cm above the floor. Each mouse was gently placed on the central platform facing an open arm, with its body orientation deliberately positioned away from the experimenter to reduce human interference. Locomotor activity was conducted for 5 min under video recording via camera. To eliminate olfactory cues between trials, the maze surfaces were thoroughly cleaned with water and dried with wipes after each subject. For the experimental data analysis, the final results were determined through dual independent scoring conducted by blinded researchers to ensure unbiased quantification.

### Tail suspension test (TST)

Depressive-like behavior was assessed by TST. In brief, mice were suspended by the tail on a horizontal bar (at around 40 cm from the floor) with adhesive tape, and immobility time was recorded during the last 4 min of the 6 min test. Mice were considered motionless when they did not exhibit escape-oriented behavior and passively hung without any body movements.

### Contextual and cued fear conditioning test

Before all experiments, mice were transported to the behavior room and left at least 30 min to acclimate. Individual mouse was placed in a test chamber and received five pairings of conditioned stimulus (30 sec of tone at 85 dB and sound frequency of 5,000 Hz) and unconditioned stimulus (interstimulus interval, 2 min) for fear conditioning. Conditioned stimulus terminated together with a foot shock (2 sec and 0.65 mA) for fear acquisition. The next day, each mouse was placed in the same chamber without any conditional stimulus for 5 min for the contextual tests. After a 2-hour break, each mouse was placed in a modified chamber for a 3-min acclimation period, followed by a 1-min auditory stimulus (85 dB and 5,000 Hz). Tone-cued test data were record for 3 min after sound stimulus. Four days later, the same experiment was repeated for fear extinction test.

### Neural circuit tracing

To comprehensively illustrate LS connectivity patterns, double co-injections were made into the 1) rostral, 2) caudal-dorsal, 3) caudal-intermediate, or 4) caudal-ventral LS. Mice were initially anesthetized with isoflurane and subsequently mounted to the stereotaxic apparatus where they were maintained under anesthetic state. Tracers were delivered iontophoretically using glass micropipettes (O.D. 28-32 µm). A positive 5 µAmp, 7 sec alternating current was delivered for 10 min. Buprenorphine (0.05 mg/kg), an opioid analgesic, was administered via subcutaneous injection once daily on the day of surgery and postoperative day 1 to manage pain. PHAL (2.5%; Vector Laboratories) was co-injected with CTb (647 conjugate, 0.25%; Invitrogen), while AAV (td-tomato; 2×10^13^ GC/mL; Addgene) and FG (1%; Fluorochrome LLC) were injected in combination. Animals were sacrificed with an overdose injection of pentobarbital (6 mg/kg) 7 days following surgeries. Each animal was then trans-cardially perfused with approximately 25 ml of 0.9% NaCl followed by 25 ml of 4% paraformaldehyde solution (PFA; pH 9.5). Brains were post-fixed in 4% PFA for 24 h at 4°C after which they were embedded in 3% Type I-B agarose and sectioned into four series of coronal sections at 50 µm thickness. One of four series was stained for PHAL using the free-floating method. Briefly, sections were transferred to a blocking solution for 1 h. Following three rinses, sections were incubated with 1:1000 concentration of rabbit anti-PHAL antibody for 48 h at 4°C along with blocking solution. Sections were rinsed three times in T-PBS and then soaked for 3 h in the secondary antibody solution (1:1000 concentration of anti-rabbit IgG conjugated with Alexa Fluor 488). Sections were counterstained with the fluorescent Nissl stain NeuroTrace 435/455 (1:1000), mounted, and cover slipped using 65% glycerol. Sections were imaged using an Olympus VS110 virtual slide scanner.

### Real-Time Quantitative Reverse Transcription PCR (qRT-PCR)

Following extraction of total RNA of LS, qRT-PCR was performed to detect the expression of mRNA. Fold changes relative to GAPDH were calculated by 2^-ΔΔCt^ and expressed as means ± standard error of means (SEM). The primer sequence is as follows (5′-3′):

m-*Mbp*-F: GGCGGTGACAGACTCCAAG

m-*Mbp*-R: GAAGCTCGTCGGACTCTGAG

m-*Ivd*-F: GGACGGCGAGTTTCCAGTT

m-*Ivd*-R: CTCCTCGTTTAGCCCGTTGA

m-*Sorl1*-F: AGCAGGAGGGAGTCGAGAC

m-*Sorl1*-R: GTTCCTAGCCGGAGATCGC

m-*Ndn*-F: GAGGTCCCCGACTGTGAGAT

m-*Ndn*-R: TGCAGGATTTTAGGGTCAACATC

m-*Grm7*-F: ACACGGATCGCAAATGCAC

m-*Grm7*-R: CTCCCCGGTAGTCAGCACA

m-*Glp1r*-F: ACGGTGTCCCTCTCAGAGAC

m-*Glp1r*-R: ATCAAAGGTCCGGTTGCAGAA

m-*Ndrg4*-F: GACATCGAGACGCCTTATGGA

m-*Ndrg4*-R: GTGATTGAGACCCACATCATGG

m-*Synpo*-F: CCTGCCCGTAACTTCCGTG

m-*Synpo*-R: GAGCGGCGGTAGGGAAAAG

m-*Tafa2*-F: TAGTGACCTTGTGGGGGAAAG

m-*Tafa2*-R: TTTGGGACCGTTCTTCTATCTTG

m-*Gapdh*-F: AGGTCGGTGTGAACGGATTTG

m-*Gapdh*-R: TGTAGACCATGTAGTTGAGGTCA

### Statistical analysis

All data were analyzed and plotted as means ± SEM using GraphPad Prism9. A Student's t test was used to compare parametric data and the Mann-Whitney test was used for non-parametric data. For multiple-group comparison, data were analyzed with one- or two-way ANOVA analysis. For multiple time point comparisons, data were assessed with repeated measures ANOVA. In all tests, *p* < 0.05 was considered a statistically significant difference.

## Supplementary Material

Supplementary figures.

## Abbreviations

LS: lateral septum
MERFISH: multiplexed error-robust fluorescence *in situ* hybridization
ESR1: estrogen receptor α
snRNA-seq: single-nucleus RNA sequencing
MS: medial septum
PTSD: post-traumatic stress disorder
LSd: dorsal lateral septum
LSi: intermediate lateral septum
LSv: ventral lateral septum
LSr: rostral lateral septum
SST: somatostatin
CRHR2: corticotropin-releasing hormone receptor 2
AHN: anterior hypothalamic nucleus
CA1.d: dorsal CA1
CA3.d: dorsal CA3
OLs: oligodendrocytes
OPCs: oligodendrocyte precursor cells
DEGs: differentially expressed genes
GSVA: Gene Set Variation Analysis
GSEA: Gene Set Enrichment Analysis
STR: striatum
LSNs: lateral septum neuronal subclusters
LSXN: lateral septum complex neuronal subcluster
LSMN: neuronal subclusters localized at the interface between the LS and adjacent brain regions
Neg. Reg. of tRS/T-K: Negative regulation of transmembrane receptor protein serine/threonine kinase
PPI: protein-protein interaction
ISH: *in situ* hybridization
CA1.v: ventral CA1
AON: anterior olfactory nucleus
PAG: periaqueductal gray
CA3.v: ventral CA3
VTA: ventral tegmental area
MEA: medial amygdala
PV: parvalbumin
BNSTpr: bed nuclei of the stria terminalis, posterior division, principal nucleus
MPO: medial preoptic area
VMHvl: ventromedial hypothalamic nucleus, ventrolateral part
GWAS: genome-wide association studies
OVA: ovalbumin
HPA: hypothalamic-pituitary-adrenal
RPCA: Robust Principal Component Analysis
UMAP: Uniform Manifold Approximation and Projection
KEGG: Kyoto Encyclopedia of Genes and Genomes
CCA: canonical correlation analysis
OFT: open field test
EPM: elevated plus maze test
TST: tail suspension test
PFA: paraformaldehyde
