# Supplementary material for: Spatial Cell Atlas of Lateral Septum Reveals Changes Underlying Anxiety and Fear Learning Deficits in Mice with Abnormal Immunity
Source: Int J Biol Sci. 2025 Oct 1;21(14):6389–410. doi: 10.7150/ijbs.117249 (PMC12594593; doi:10.7150/ijbs.117249)
Supplement: Supplementary file 1 — Supplementary figures. [file ijbsv21p6389s1.pdf]

Supplementary materials

Spatial Cell Atlas of Lateral Septum Reveals Changes Underlying Anxiety and Fear Learning Deficits in Mice with Abnormal Immunity

Yian Wang[1,†], Wenxia Gao[1,†], Xueyan Yang[1], Zirui Liu[1], Hisham Al-Ward[1], Orion R Fan[1], Yiming Shao[1], Liqiang Zhou[2], Bo Jing[3], Qianxiang Wu[1], Wenmin Zhu[2,✉], Wei Chen[1,✉], Yi Eve Sun[1,✉]

1. Stem Cell Translational Research Center, Tongji Hospital, School of Medicine, Tongji University, Shanghai, 200065, China
2. Faculty of Life and Health Sciences, Shenzhen University of Advanced Technology (SUAT), Shenzhen, Guangdong 518000, China
3. Brain Cognition and Brain Disease Institute, Shenzhen Institutes of Advanced Technology, Chinese Academy of Sciences, Shenzhen 518055, China

† Equal contribution

✉ Corresponding authors:
Wenmin Zhu: zwm8899@163.com. Wei Chen: oliverchennemocw@163.com. Yi Eve Sun: yi.eve.sun@gmail.com and sunyi2013@tongji.edu.cn.

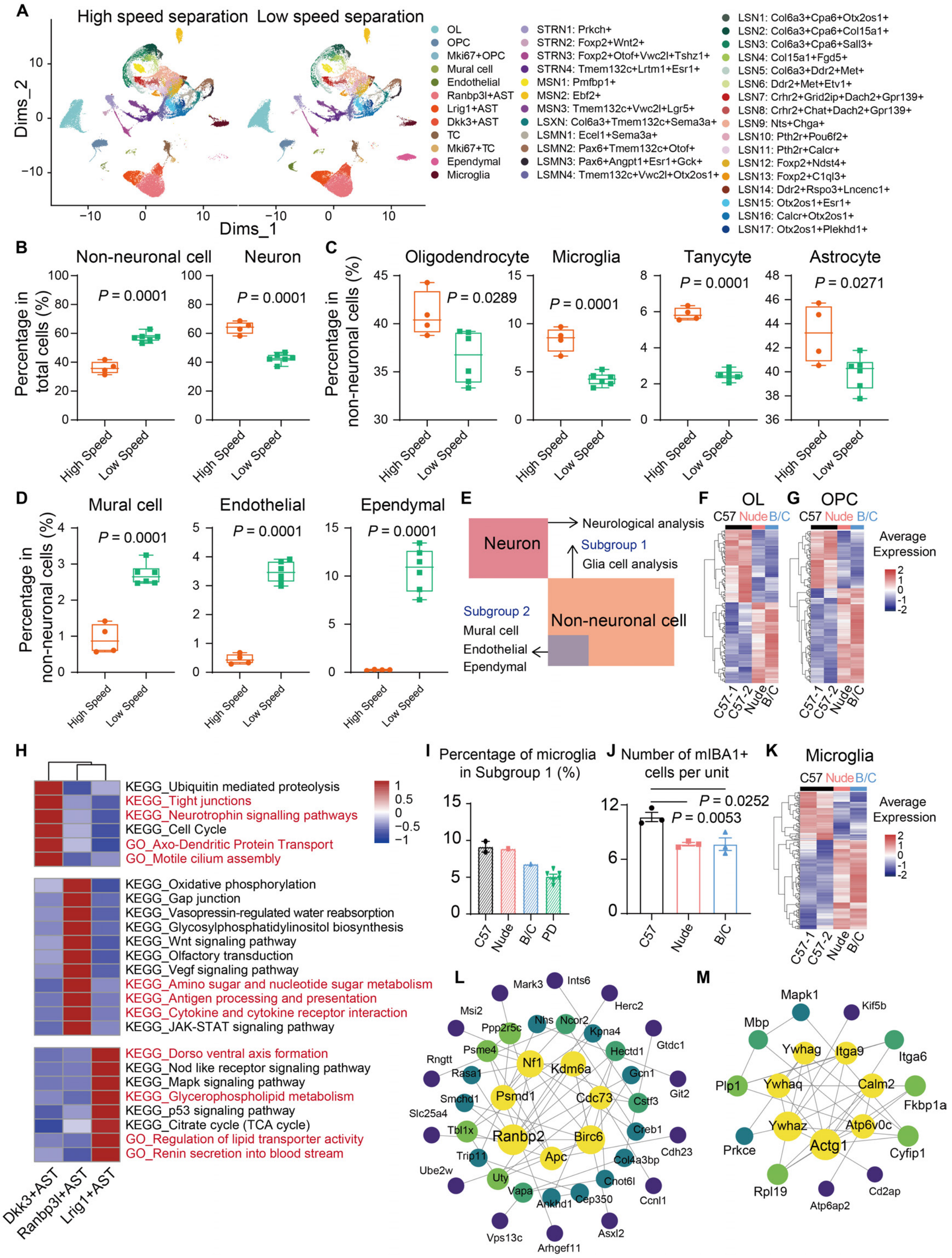

Fig. S1 The analysis logic of snRNA-seq and DEG analysis of non-neuronal cells. A The UMAP plots of all cell clusters collected, showing the similarities and differences between our data (high speed separation) and the public data (low speed separation). **B** Comparison of the proportions of non-neuronal cells and neuron between two separation methods (Median ± upper and lower quartiles, High speed, n = 4; Low speed, n = 6; Student's t test was used). **C, D** Specific non-neuronal subtypes bias analysis (Median ± upper and lower quartiles, High speed, n = 4; Low speed, n = 6; Student's t test was used). **E** Schematics overview of subsequent analysis. **F, G** Heatmap of DEGs of OL and OPC

between C57J mice cohort and nude, BALB/c mice cohort. **H** The heatmap illustrates the enrichment terms calculated by GSVA across astrocyte subclusters. **I** Percentage of microglia across three mouse strains based on snRNA-seq data. **J** Quantitative analysis of the number of IBA1-positive cells per unit (mean ± SEM, n = 3, Student's t test was used) based on the immunofluorescence staining. **K** Heatmap of DEGs of microglia between C57J mice cohort and nude, BALB/c mice cohort. **L, M** PPI network of upregulated genes (**L**) and downregulated genes (**M**) in microglia of nude and BALB/c mice cohort, compared to C57J mice cohort.

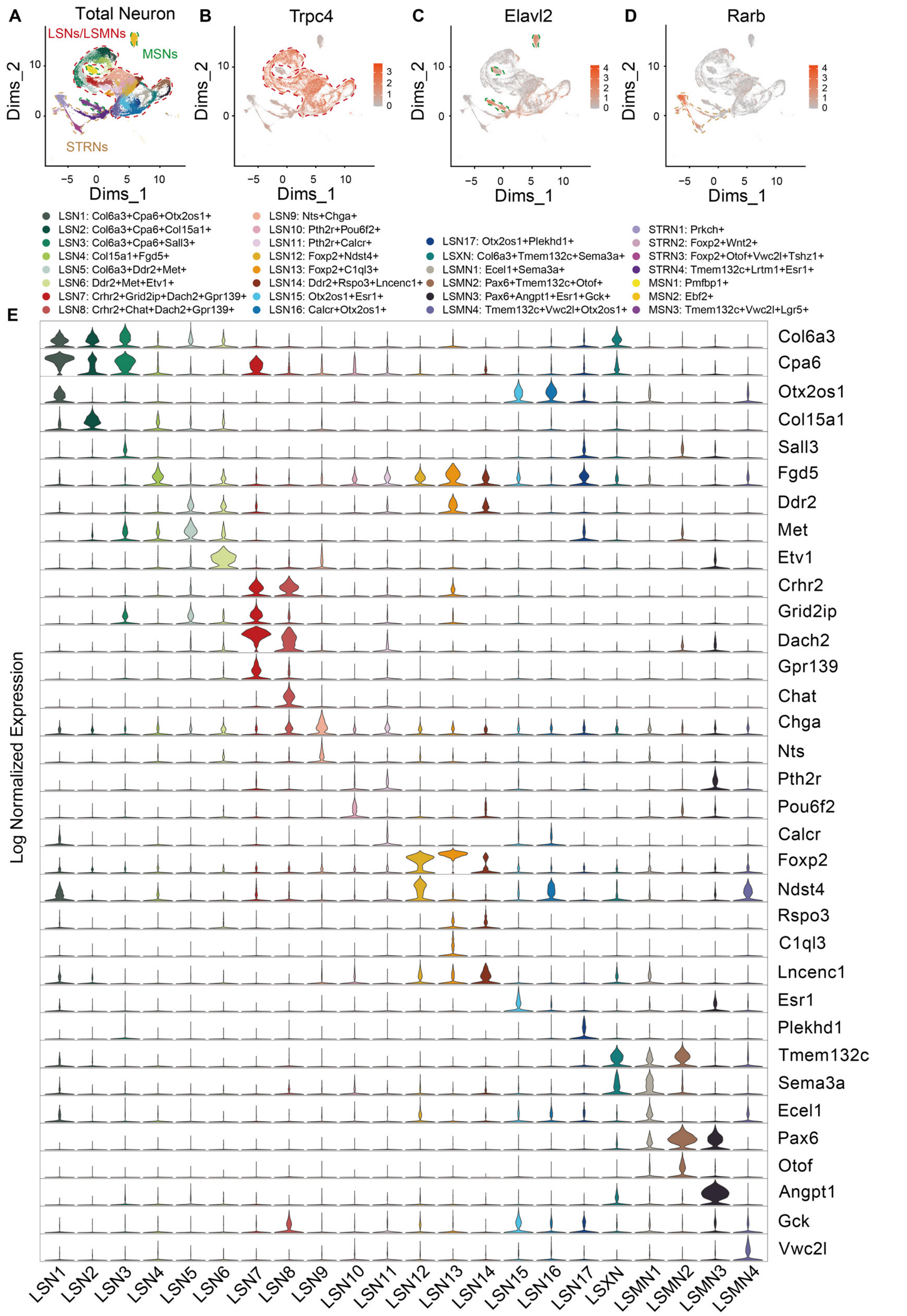

Fig. S2 Marker genes of cell clusters. A UMAP plots of total neurons. **B-D** Feature plots demonstrate brain region specificity neurons of LS (**B**), MS (**C**) and striatum (**D**). **E** The stacked violin plot displays the expression patterns of signature genes in distinct LS associated neurons.

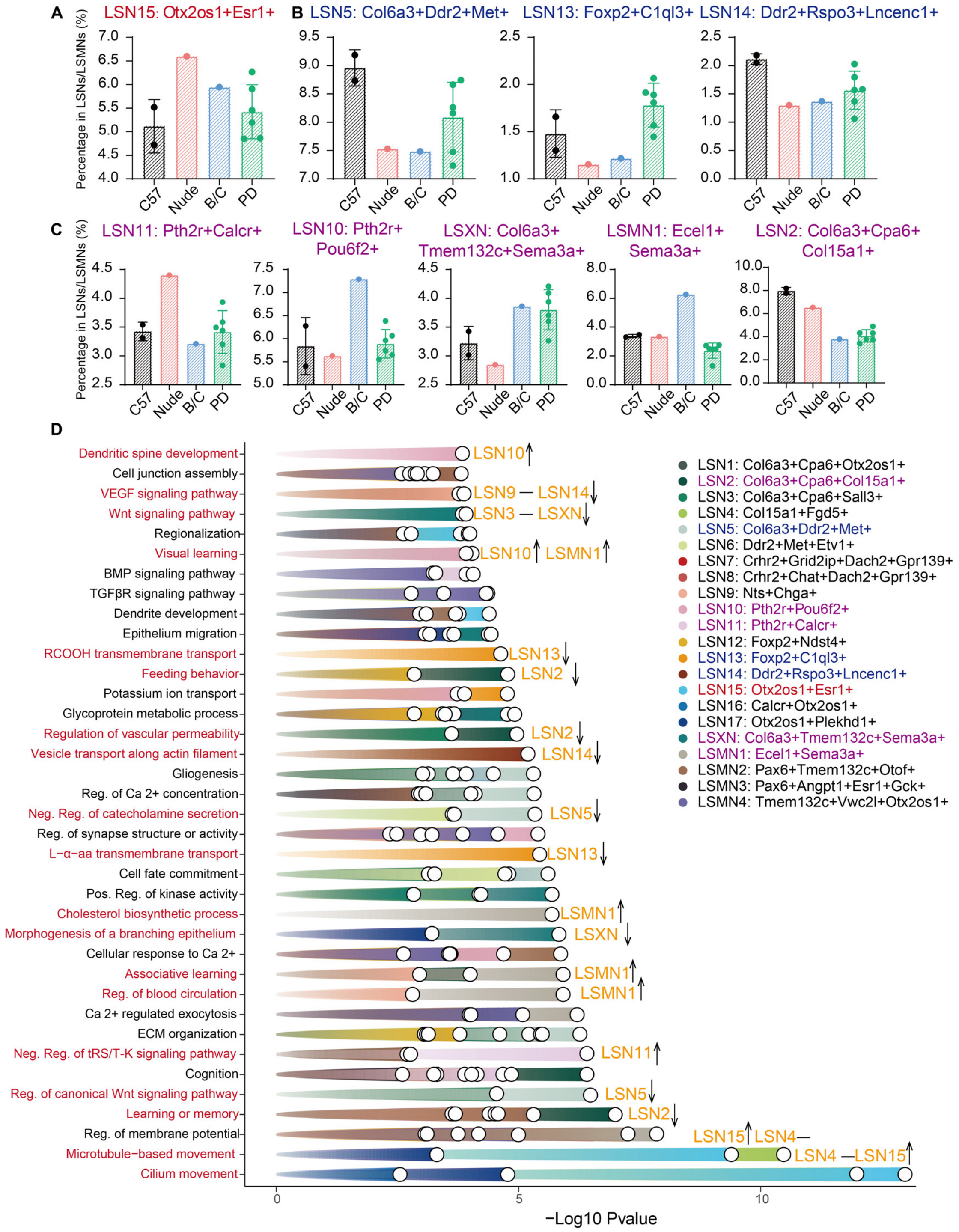

Fig. S3 Differences in neurons proportion and functional annotations. A Compared to C57J, LSNs increased in both nude and BALB/c mice. **B** LSNs decreased in both nude and BALB/c mice. **C** LSNs with specific changes in nude or BALB/c mice. **D** GO analysis revealed enriched biological process in total LS associated neurons. The terms marked in red indicate that they have LSNs specificity rather than global function. A solid arrow indicates that the proportion of these LS associated neurons changes in nude and/or BALB/c mice.

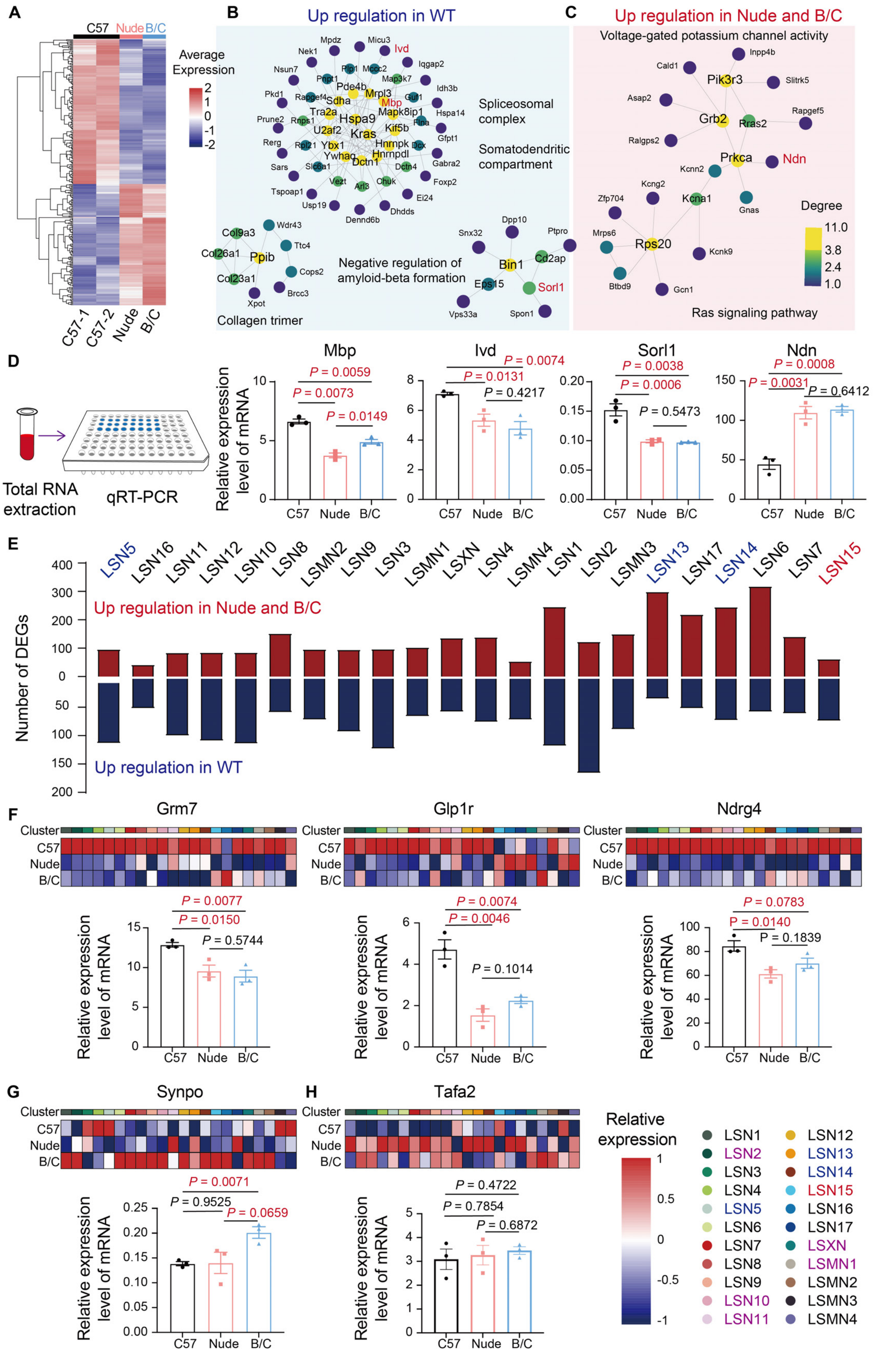

Fig. S4 DEG analyses of LS associated neurons. A Heatmap of LS associated neuron DEGs between C57J mice cohort and nude, BALB/c mice cohort. **B, C** PPI network of downregulated genes (**B**) and upregulated genes (**C**) both in nude and BALB/c mice. **D**

The left shows the schematic of qRT-PCR of mouse LS, and the right shows the mRNA expression level across three strains (mean ± SEM, n = 3, Student's t test was used). **E** Bar chart of the number of DEGs in specific LS associated neurons (total 22 neuronal subtypes). **F-H** The top heatmap shows the sequencing gene expression levels in LSNs across the three mouse strains, and the bottom plot shows the mRNA expression levels verified by qRT-PCR across three strains (mean ± SEM, n = 3, Student's t test was used).

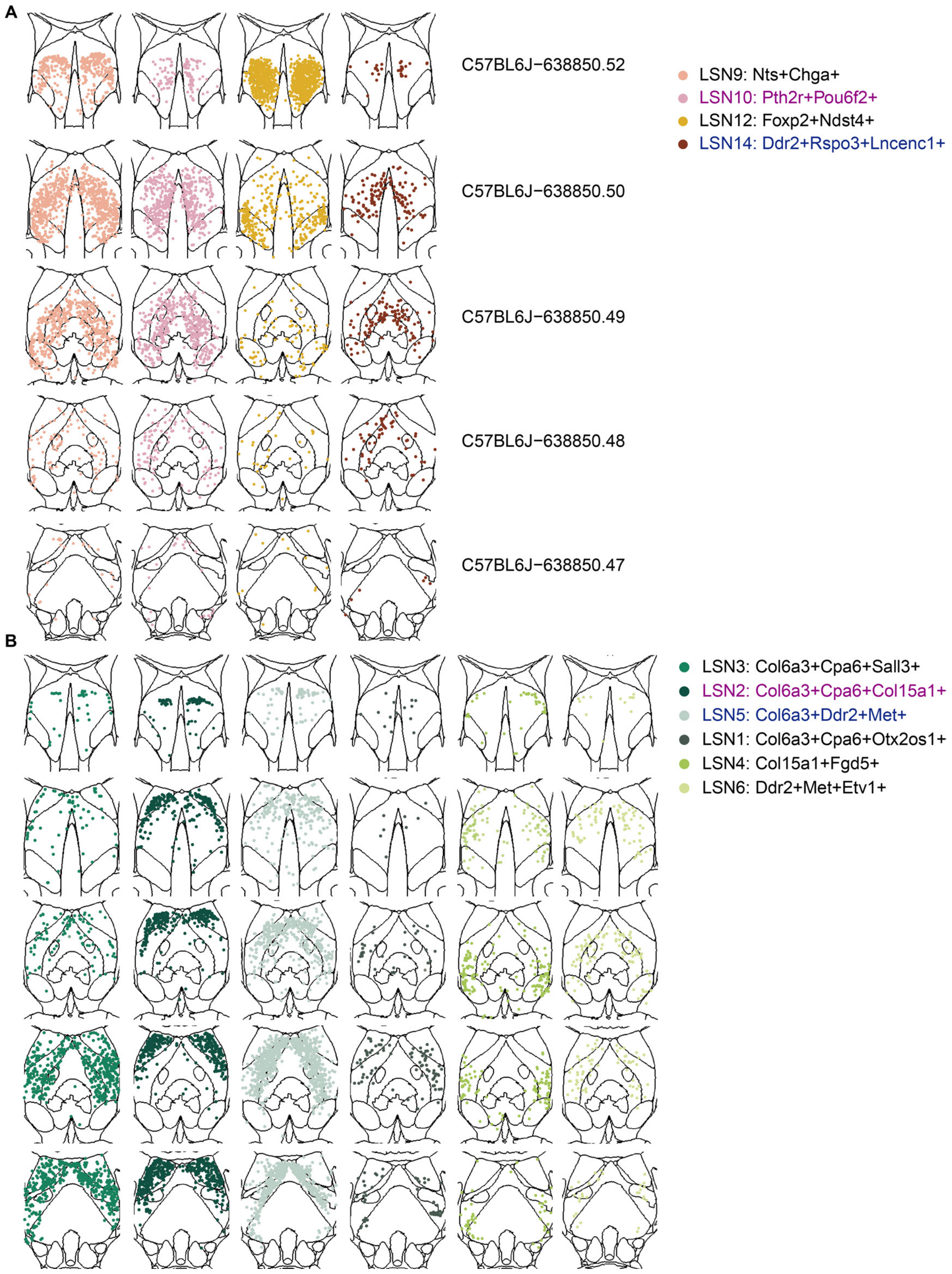

Fig. S5 Integrations of defined LS associated neurons from snRNA-seq and MERFISH spatial data. Five representative coronal sections were selected for illustrated. The rostral distribution LSNs (**A**) and caudal-dorsal distribution LSNs (**B**) were demonstrated, respectively.

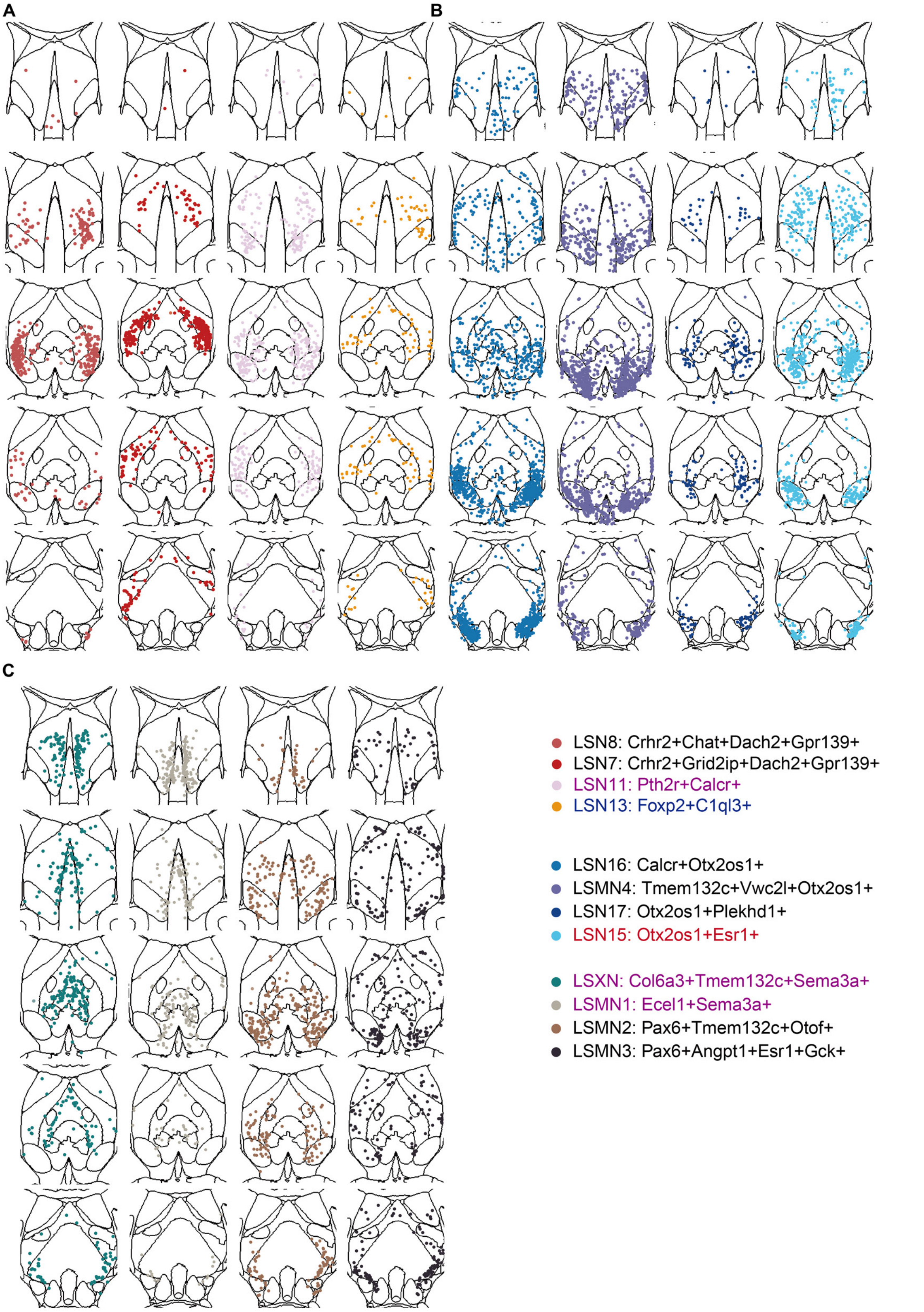

Fig. S6 Integrations of defined LS associated neurons from snRNA-seq and MERFISH spatial data. Five representative coronal sections were selected for illustrated. The caudal-intermediate distribution LSNs (**A**), caudal-ventral distribution LSNs (**B**) and neurons located at the junction of LS and other brain regions (**C**) were demonstrated, respectively.

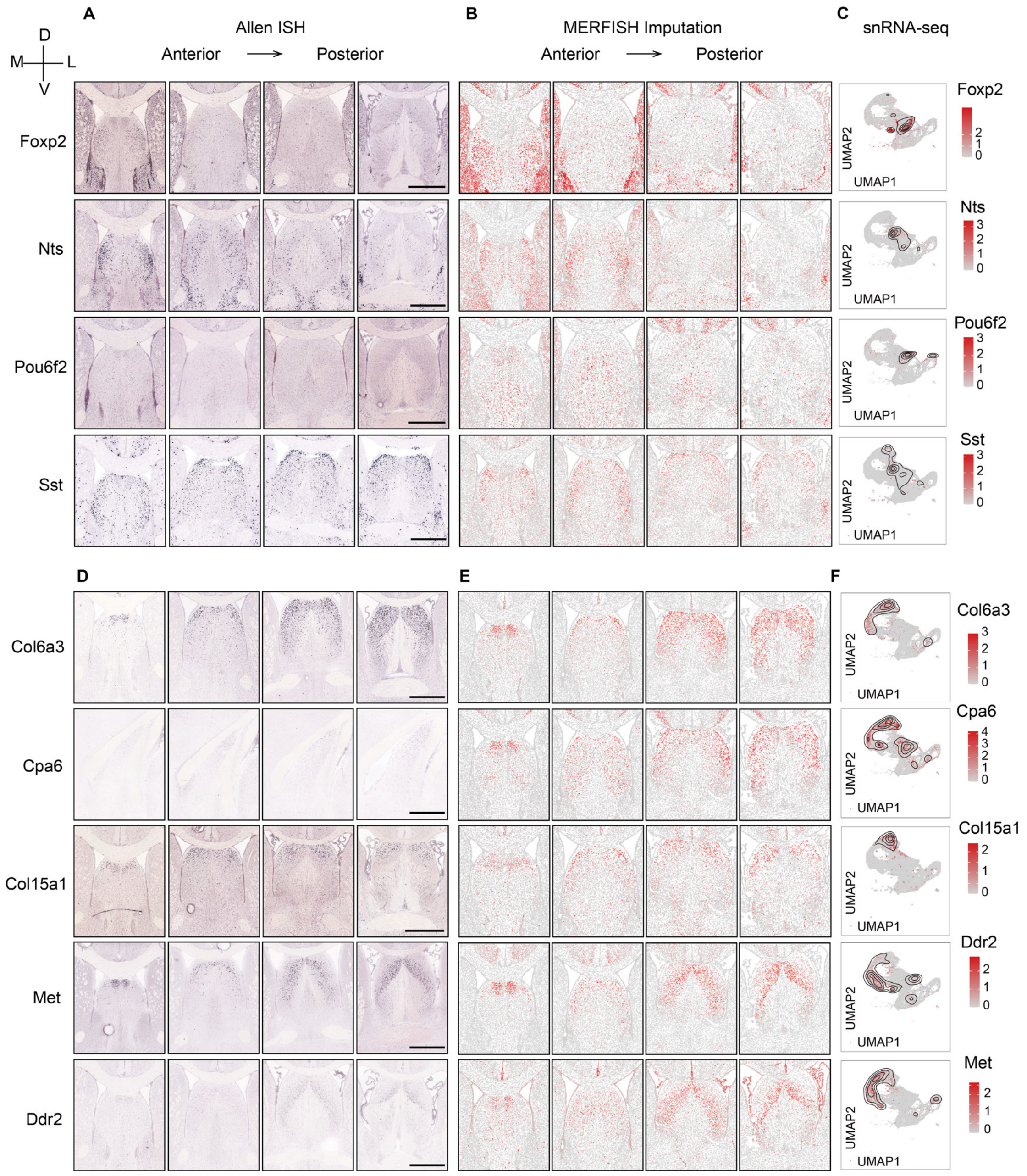

Fig. S7 Spatial gene-expression patterns to identify the LS subregion. The expression patterns of marker genes in LSNs with spatially specific distribution characteristics in in situ hybridization (ISH) data from the Allen Brain Atlas (**A, D**), imputation results (**B, E**), and subcluster specific distribution in snRNA-seq UMAP (**C, F**). **A-C** show rostral LS subregion enrichment. **D-F** show caudal-dorsal LS subregion enrichment.

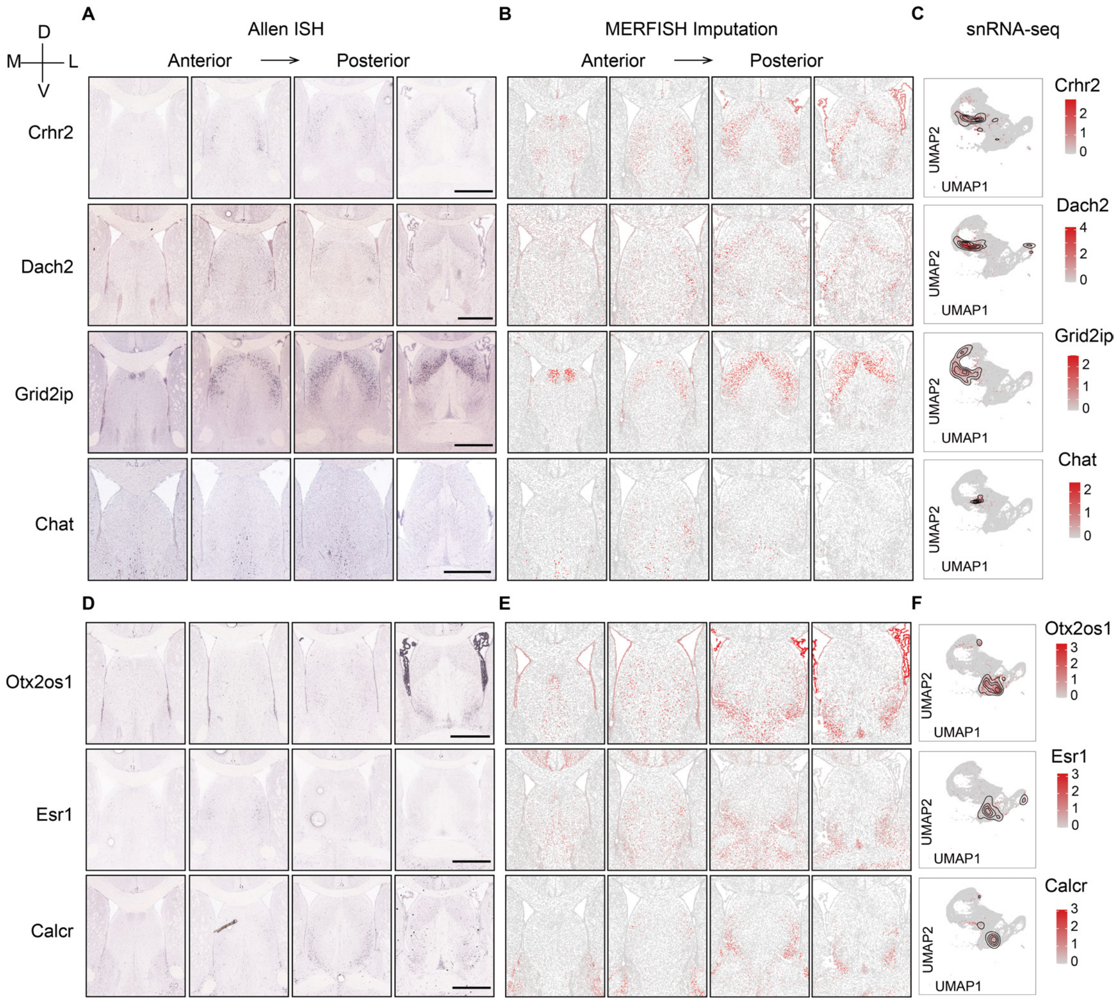

Fig. S8 Spatial gene-expression patterns to identify the LS subregion. The expression patterns of marker genes in LSNs with spatially specific distribution characteristics in in situ hybridization (ISH) data from the Allen brain atlas (**A, D**), imputation results (**B, E**), and subcluster specific distribution in snRNA-seq UMAP (**C, F**). **A-C** show caudal-intermediate LS subregion enrichment. **D-F** show caudal-ventral LS subregion enrichment.

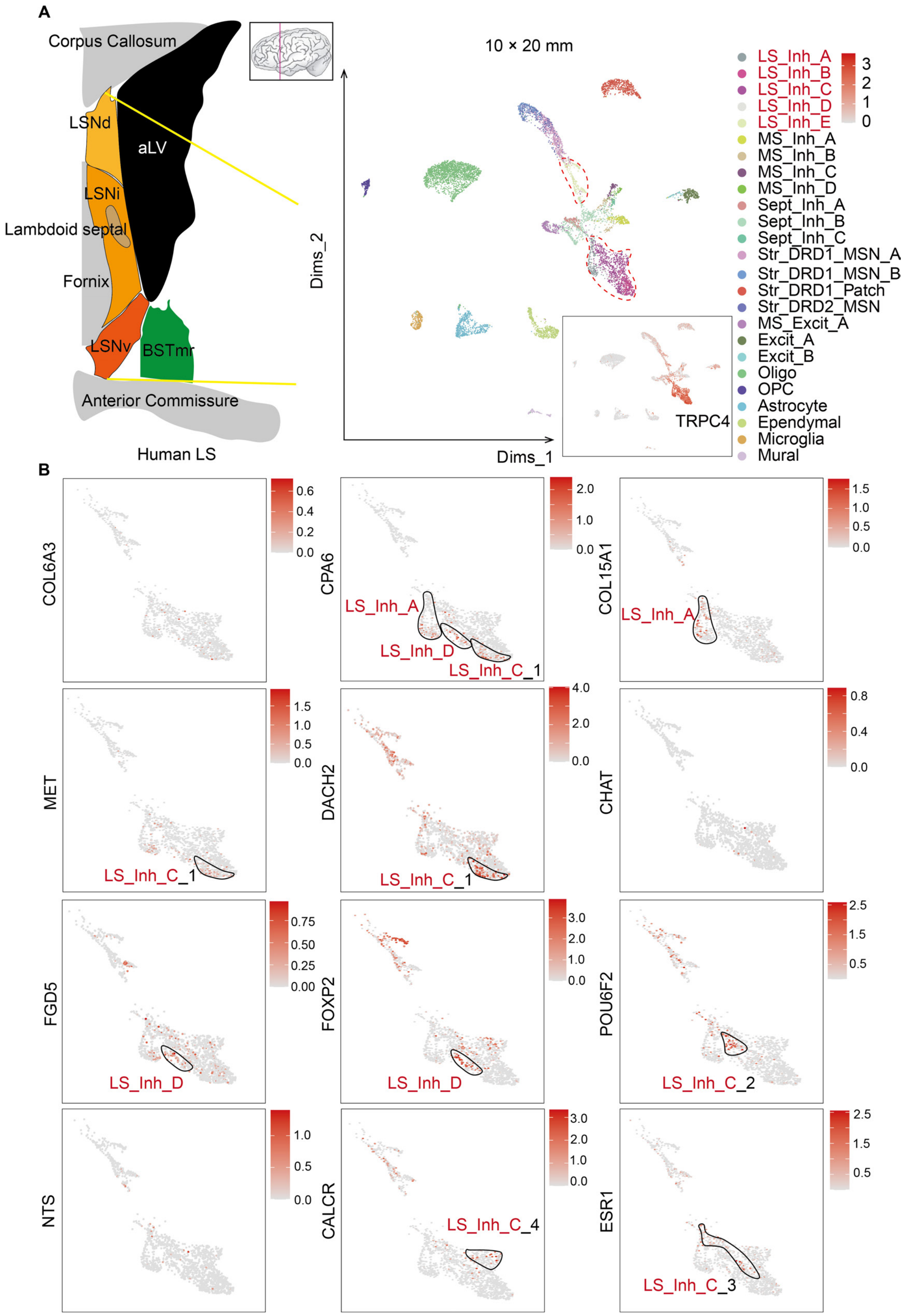

Fig. S9 Evolutionary conservation of key LSNs in mice and humans. A The left shows the schematic illustration of LS region sampling in the human brain. The right shows the UMAP plots of the collected human cells. Based on the distribution of the *TRAP4* gene, the red-highlighted legend denotes neuronal subtypes within the LS region. **B** Features plots demonstrate the expression distribution of mouse-derived LSN subtype-specific

markers in human LS dataset.

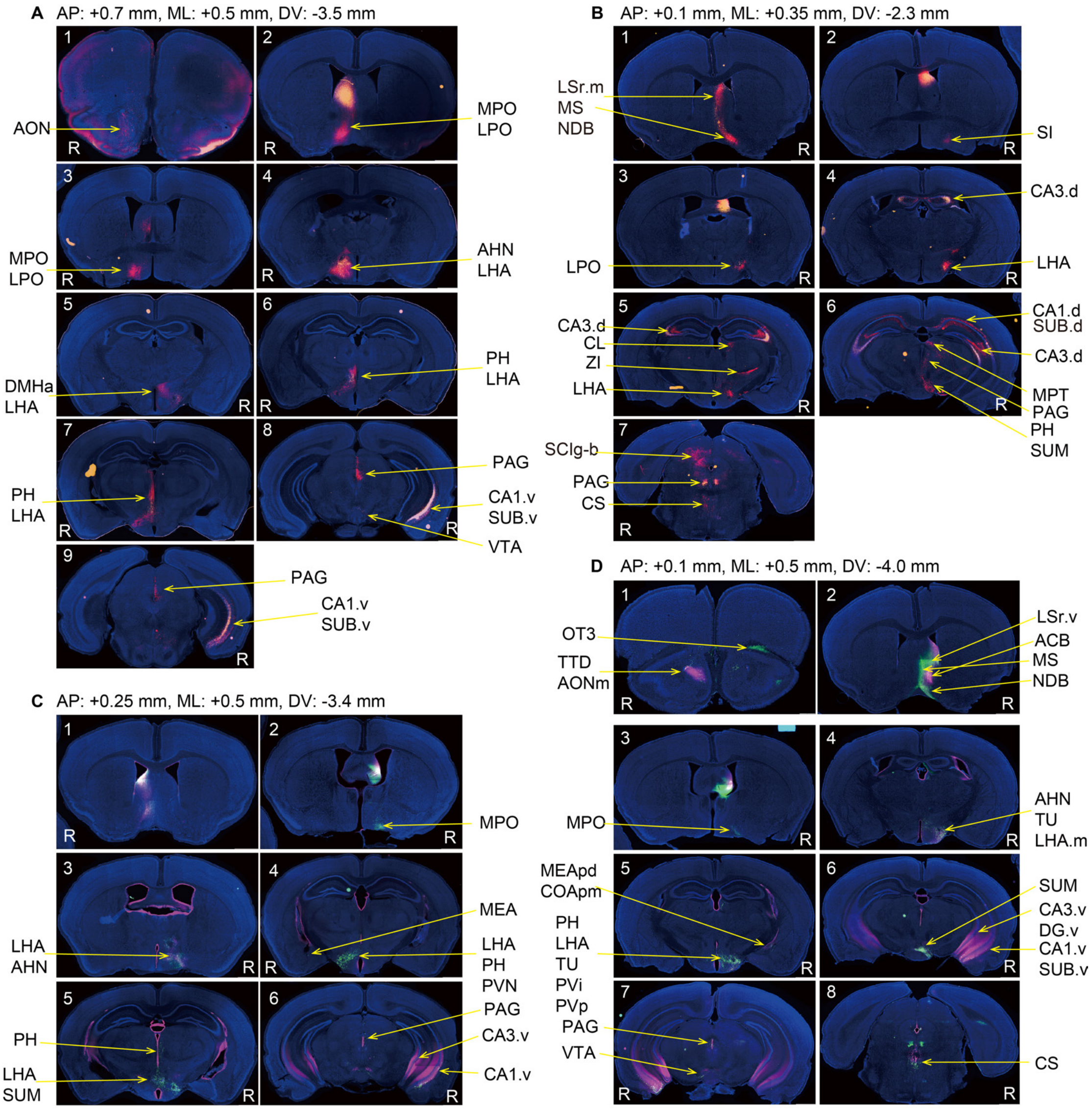

Fig. S10 Space specific projection network. A Projections network of rostral LS subregion along a series of coronal sections (yellow: input regions; red: output regions). **B** Projections network of caudal-dorsal LS subregion along a series of coronal sections (yellow: input regions; red: output regions). **C** Projections network of caudal-intermediate LS subregion along a series of coronal sections (magenta: input regions; green: output regions). **D** Projections network of caudal-ventral LS subregion along a series of coronal sections (magenta: input regions; green: output regions). The top of each section has detail coordinate information of the injection site. The R-label means the right hemisphere of the mouse brain.

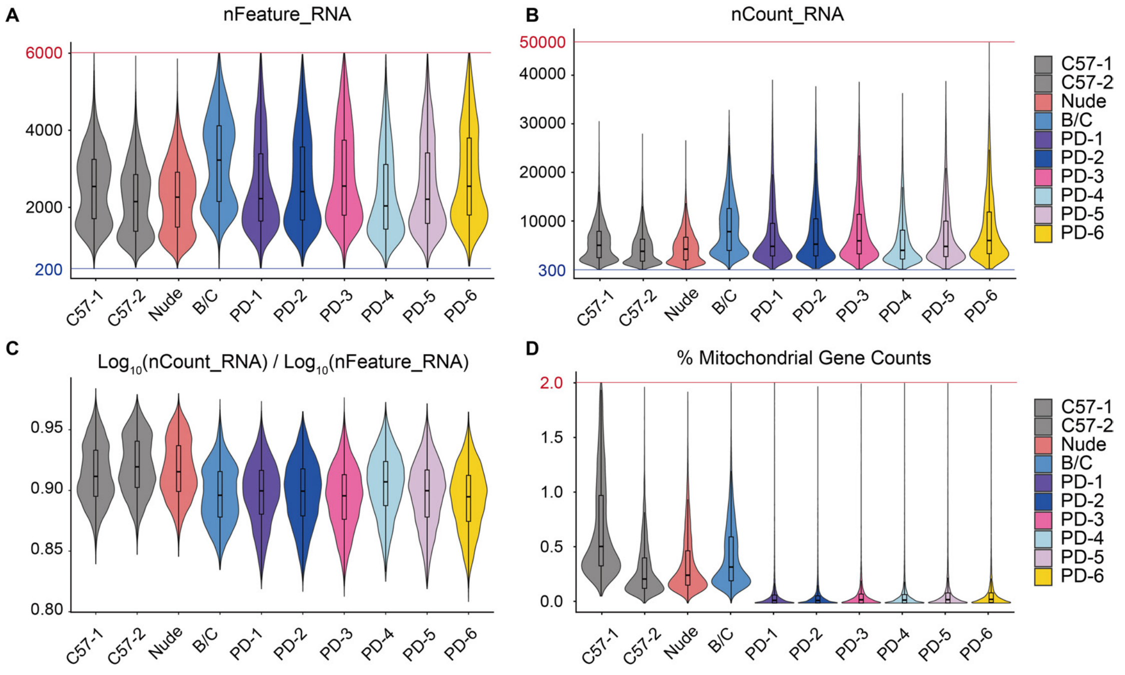

E

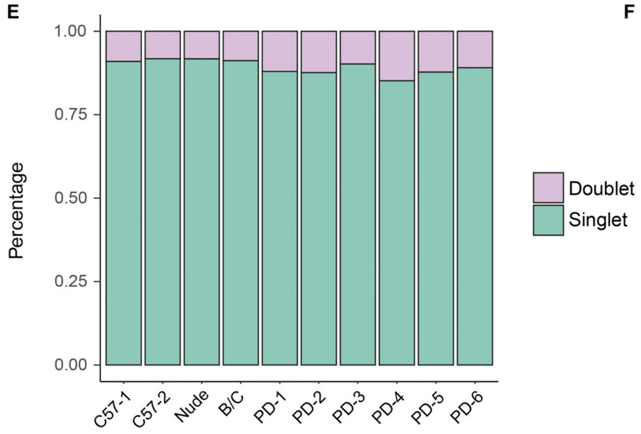

F

Sample	Singlet	Doublet
C57-1	90.95%	9.05%
C57-2	91.77%	8.23%
Nude	91.72%	8.28%
B/C	91.18%	8.82%
PD-1	87.96%	12.04%
PD-2	87.62%	12.38%
PD-3	90.18%	9.82%
PD-4	85.16%	14.84%
PD-5	87.77%	12.23%
PD-6	89.09%	10.91%

Fig. S11 Quality control of snRNA-seq data. The violin plots display the distribution of the number of **A**, detected genes per sample (nFeature_RNA), **B**, total UMI counts per sample (nCount_RNA), **C**, the ratio of log_{10}(nCount_RNA) to log_{10}(nFeature_RNA), and **D**, the percentage of mitochondrial gene counts per sample. The red line represents the upper limit of the standard, and the blue line represents the lower limit of the standard. **E, F** The overlaid bar chart illustrates the proportion of doublets in each sample, while the accompanying table provides the exact doublet percentages for each individual sample.

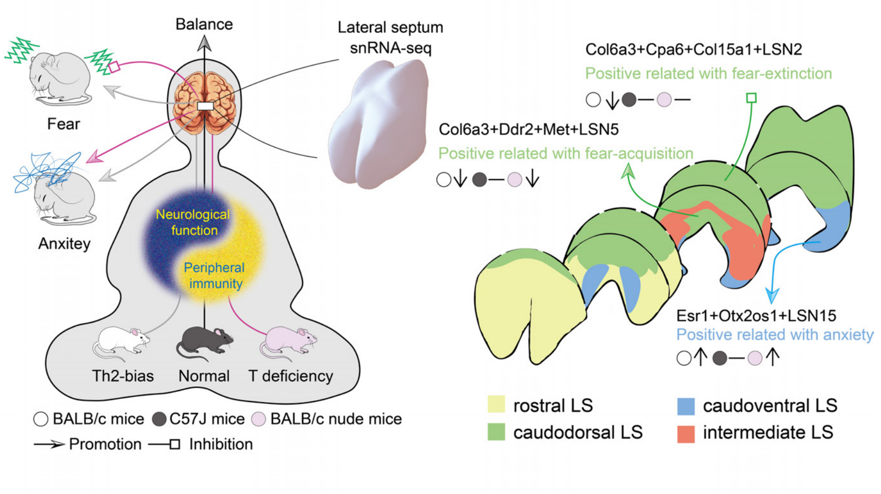

Fig. S12 Graphical abstract.
